# Radiotherapy Utilization in Traditional Medicare and Medicare Advantage

**DOI:** 10.1001/jamanetworkopen.2025.3018

**Published:** 2025-04-02

**Authors:** Jacob Hogan, E. John Orav, Tianfeng Zhang, Alexander Spektor, Jie Zheng, Thomas C. Tsai, Miranda B. Lam

**Affiliations:** 1 Harvard Radiation Oncology Residency Program, Boston, Massachusetts; 2 Department of Medicine, Brigham and Women’s Hospital, Harvard Medical School, Boston, Massachusetts; 3 Department of Radiation Oncology, Brigham and Women’s Hospital and Dana Farber Cancer Institute, Harvard Medical School, Boston, Massachusetts; 4 Department of Health Policy and Management, Harvard T.H. Chan School of Public Health, Boston, Massachusetts; 5 Department of Surgery, Brigham and Women’s Hospital, Harvard Medical School, Boston, Massachusetts

## Abstract

**Importance:**

As more than 50% of Medicare beneficiaries are enrolled in Medicare Advantage (MA), understanding whether the treatment covered by MA vs traditional Medicare (TM) is comparable can aid in providing high-value care. As the majority of patients with cancer undergo radiotherapy, it is important to quantify TM and MA utilization in oncology.

**Objective:**

To analyze the primary type of radiotherapy technology used, treatment length, and estimated spending for MA patients with cancer undergoing radiotherapy compared with TM patients with cancer.

**Design, Setting, and Participants:**

This retrospective cross-sectional study used 2018 Medicare claims data for TM and MA patients aged 65 years or older who received radiotherapy for 1 of 15 cancer types. Analyses were performed between May 1 and December 28, 2024.

**Exposures:**

Insurance type (MA vs TM), cancer type, age, dual-eligibility status, medical comorbidities, county, and radiotherapy center type.

**Main Outcomes and Measures:**

Primary type of radiotherapy technology used, treatment length, and estimated spending for 90-day radiotherapy episodes. Adjusted rates and odds ratios (ORs) were calculated to compare technology types and rate ratios (RRs) to compare treatment length and estimated spending between TM and MA episodes.

**Results:**

Of 31 563 treatment episodes among 30 941 patients, 22 594 (71.58%) were covered by TM (mean [SD] age, 74.76 [6.57] years; 50.76% among males) and 8969 (28.42%) were covered by MA (mean [SD] age, 74.51 [6.24] years; 51.78% among males). For radiotherapy episodes in patients with MA, adjusted analyses revealed lower odds of proton therapy use (52 [0.58% (95% CI, 0.34%-0.82%)] vs 373 [1.65% (95% CI, 1.50%-1.80%)]; OR, 0.36 [95% CI, 0.27-0.48]) and stereotactic radiotherapy use (1235 [13.77% (95% CI, 13.13%-14.41%)] vs 3391 [15.01% (95% CI, 14.61%-15.41%)]; OR, 0.87 [95% CI, 0.81-0.95]), higher odds of 2- or 3-dimensional radiotherapy use (3962 [44.17% (95% CI, 43.39%-44.96%)] vs 9584 [42.43% (95% CI, 41.93%-42.92%)]; OR, 1.13 [95% CI, 1.06-1.21]), greater mean treatment length (21.38 [95% CI, 21.14-21.61] vs 19.48 [95% CI, 19.33-19.62] treatments; RR, 1.10 [95% CI, 1.08-1.11]), and higher estimated radiotherapy spending ($8677.56 [95% CI, $8566.58-$8788.54] vs $8393.20 [95% CI, $8323.34-$8463.05]; RR, 1.04 [95% CI, 1.02-1.06]) compared with episodes in patients with TM.

**Conclusions and Relevance:**

In this cross-sectional study, MA patients with cancer undergoing radiotherapy had higher estimated spending and greater mean treatment length than those covered by TM. Despite lower utilization of more expensive advanced treatment modalities, MA was not associated with cost savings. Whether MA meets the value proposition for radiation oncology requires further investigation.

## Key Points

**Question:**

Are there differences in utilization of radiotherapy technologies, treatment length, and estimated spending between patients with cancer enrolled in Medicare Advantage (MA) plans and those with traditional Medicare (TM)?

**Findings:**

In this cross-sectional study of 31 563 radiotherapy episodes among 30 941 patients, for episodes in patients with MA vs TM, significantly lower odds of proton and stereotactic radiotherapy use, higher odds of 2- or 3-dimensional radiotherapy use, longer mean treatment length, and higher estimated radiotherapy spending were found.

**Meaning:**

These results suggest that use of expensive, advanced-technology treatments for cancer may be decreased among patients with MA vs those with TM, but radiotherapy utilization and estimated cost may be increased.

## Teaser

This cross-sectional study investigates whether differences in radiotherapy technologies, treatment length, and spending exist between traditional Medicare and Medicare Advantage beneficiaries with cancer.

## Introduction

Ensuring access to affordable and high-quality health care for aging individuals is crucial. For older adults in the US, traditional Medicare (TM) and Medicare Advantage (MA) represent 2 primary pathways to access health care, each with distinct pros and cons. Traditional Medicare includes Part A (inpatient) and Part B (outpatient) services, allowing patients to receive care nationwide from any physician accepting Medicare, without preauthorization, and paying 80% of qualifying services. However, TM lacks an out-of-pocket maximum, often necessitating additional Medigap (Medicare supplemental insurance) and Part D plans for co-payments, deductibles, and prescription drugs. In contrast, Medicare Advantage (Part C) plans are government funded but privately managed, offering comprehensive coverage with a defined out-of-pocket limit and typically lower premiums and, thus, eliminating the need for additional Medigap or Part D plans. Medicare Advantage plans may also offer additional benefits, such as gym memberships and vision or dental coverage. Yet, MA plans may feature limited practitioner networks, require preauthorization for certain services, and complicate transitions back to TM due to potential Medigap enrollment challenges.^1,2^

Despite these differences, MA enrollment has surged, partially driven by cost benefits but also potential factors like aggressive marketing and higher broker commissions.^3,4^ Notably, studies indicate that patients with functional disabilities or high needs often switch back to TM, suggesting gaps in MA coverage for complex cases.^5,6^ For patients with cancer, who typically face complex health care, assessing whether MA delivers on its promise of efficient, high-quality care is essential. While MA demonstrates potential advantages for patients, such as lower monthly premiums and enhanced primary care access, concerns persist regarding limited networks, prior authorization, and rising out-of-pocket expenses.^3,7,8,9,10,11,12,13^ There is growing concern that efforts by MA health plans to decrease utilization may also result in lower quality of care.^14,15^

Cancer treatment frequently involves radiotherapy, which may be substantially influenced by the utilization management strategies of MA. Advanced techniques like proton therapy are often subject to authorization, creating care delays.^16,17,18^ Evidence also suggests that some MA patients with cancer lack access to high-volume, in-network hospitals.^19^ Understanding the impact of MA on radiation oncology, specifically regarding treatment technology, duration, and spending, is crucial.^19,20^ Our study aimed to address these gaps by examining differences in type of radiotherapy technology, radiotherapy length, and radiotherapy spending per 90-day episode for patients with cancer covered by MA compared with those covered by TM.

## Methods

### Dataset

In this cross-sectional study, we used Medicare claims data from a 20% random sample of 2018 TM and MA beneficiaries, with analyses performed between May 1 and December 28, 2024.^21^ We used the Outpatient and Carrier files, as most radiotherapy occurs in the outpatient setting. We included patients aged 65 years or older who received radiotherapy for 1 of the 15 cancer types included in the proposed Radiation Oncology Case Rate program.^20^ We followed the Centers for Medicare & Medicaid Services algorithm to create 90-day radiotherapy episodes, permitting multiple episodes per patient. Incomplete episodes were excluded. Consistent with previous studies, we included MA plans with highly reliable data,^22,23^ defined as those with less than 10% of hospital stays missing in the encounter data and with less than a 10% discrepancy in outpatient and emergency department visits between encounter data and Healthcare Effectiveness Data and Information Set data.^23^ This study received an exemption, with a waiver of informed consent, from the Harvard T.H. Chan School of Public Health institutional review board because risk was minimal, data were deidentified, and obtaining informed consent was not feasible. We followed the Strengthening the Reporting of Observational Studies in Epidemiology (STROBE) reporting guideline.

### Variables

The primary variable of interest was insurance status. Patients who were continuously enrolled in either MA or TM for the entire year were included. We categorized 90-day radiotherapy episodes as covered by MA (MA episodes) or TM (TM episodes). Several demographic and clinical variables were quantified: dual-eligibility status (eligibility for both Medicare and Medicaid), age, sex, racial and ethnic group, cancer type, type of radiotherapy center, death during the treatment year, and Chronic Conditions Data Warehouse comorbidities (Table 1 and eTable 1 in Supplement 1). Race and ethnicity, determined using the Research Triangle Institute variable,^24^ were analyzed to ensure that we accounted for potential systemic differences in treatment for racial and ethnic groups. Categories included African American or Black, Hispanic or Latinx, White, other (which included American Indian or Alaska Native, Asian or Pacific Islander, and other), and unknown. The *International Statistical Classification of Diseases and Related Health Problems, 10th Revision* codes used to define the 15 cancer types are listed in eTable 2 in Supplement 1.

**Table 1.** Baseline Patient Characteristics by Episode, Stratified by TM vs MA

Characteristic	Radiotherapy episodes, No. (%) (N = 31 563)		SMD
	TM (n = 22 594)	MA (n = 8969)	
Dual-eligibility status			
Dual	2606 (11.53)	1243 (13.86)	−0.0711
Nondual	19 988 (88.47)	7726 (86.14)	0.0711
Age group, y			
65-74	12 234 (54.15)	4986 (55.59)	−0.0290
75-84	8227 (36.41)	3286 (36.64)	−0.0047
≥85	2133 (9.44)	697 (7.77)	0.0585
Sex			
Female	11 125 (49.24)	4325 (48.22)	0.0203
Male	11 469 (50.76)	4644 (51.78)	−0.0203
Race and ethnicity			
African American or Black	1667 (7.38)	1235 (13.77)	−0.2223
Hispanic or Latinx	851 (3.77)	752 (8.38)	−0.2113
White	19 016 (84.16)	6603 (73.62)	0.2717
Other^a^	696 (3.08)	257 (2.87)	0.0126
Unknown	364 (1.61)	122 (1.36)	0.0204
Cancer type			
Anal	196 (0.87)	71 (0.79)	0.0083
Bladder	335 (1.48)	137 (1.53)	−0.0037
Bone metastases	3088 (13.67)	1100 (12.26)	0.0414
Brain metastases	1867 (8.26)	708 (7.89)	0.0135
Breast	4274 (18.92)	1734 (19.33)	−0.0106
CNS	414 (1.83)	117 (1.30)	0.0410
Cervical	100 (0.44)	62 (0.69)	−0.0348
Colon	551 (2.44)	224 (2.50)	−0.0038
Head and neck	1108 (4.90)	421 (4.69)	0.0098
Lung	4069 (18.01)	1580 (17.62)	0.0103
Lymphoma	701 (3.10)	252 (2.81)	0.0171
Pancreatic	314 (1.39)	115 (1.28)	0.0093
Prostate	4231 (18.73)	1922 (21.43)	−0.0683
Upper GI	622 (2.75)	233 (2.60)	0.0096
Uterine	724 (3.20)	293 (3.27)	−0.0035
Radiotherapy center type			
Freestanding^b^	8236 (36.45)	3833 (42.74)	−0.1295
Hospital^c^	13 890 (61.48)	4996 (55.70)	0.1179
Both equally^d^	468 (2.07)	140 (1.56)	0.0371
Died during treatment year	4629 (20.49)	1743 (19.43)	0.0263
Comorbidities			
COPD	5870 (25.98)	2336 (26.05)	−0.0015
CHF	3479 (15.40)	1263 (14.08)	0.0368
Diabetes	5727 (25.35)	2376 (26.49)	−0.0262
Heart arrhythmias	4383 (19.40)	1504 (16.77)	0.0676
Vascular disease	4994 (22.10)	1943 (21.66)	0.0106

Abbreviations: CHF, congestive heart failure; CNS, central nervous system; COPD, chronic obstructive pulmonary disease; GI, gastrointestinal; MA, Medicare Advantage; SMD, standardized mean difference; TM, traditional Medicare.

^a^ Included American Indian or Alaska Native, Asian or Pacific Islander, and other.

^b^ Included episodes in which most or all radiation therapy codes were in the Medicare Carrier File (indicating a freestanding radiotherapy center).

^c^ Included episodes in which most or all radiation therapy codes were in the Medicare Outpatient File (indicating a hospital-based radiotherapy center).

^d^ Included episodes in which the number of radiation therapy codes in the Medicare Carrier File (indicating a freestanding radiotherapy center) and Medicare Outpatient File (indicating a freestanding radiotherapy center) were equal.

### Outcomes

There were 3 primary outcomes: type of radiotherapy technology, radiotherapy length, and radiotherapy spending during the 90-day episode. Radiotherapy technology was categorized based on the majority of radiotherapy delivery codes for each episode, categorized into 5 types: 2- or 3-dimensional (2D-3D), intensity-modulated radiotherapy (IMRT), proton beam, stereotactic, or brachytherapy. Proton beam and stereotactic radiotherapy were considered to be advanced technologies. Episodes with an equal number of treatments using the top 2 technologies were unable to be categorized as having a single majority treatment modality and were excluded. Treatment length was defined as the total number of radiotherapy visits (regardless of technology) per 90-day episode. To address outliers, the top 0.5% of treatments for each technology type were winsorized. For spending analysis, standardized spending data were available for TM episodes and summed to determine radiotherapy spending. For MA episodes, spending data were estimated using procedure codes as if they were billed under TM using the Medicare Physician Fee Schedule. When codes lacked Medicare Physician Fee Schedule prices, mean TM reimbursement was used. Codes without pricing in both TM and MA were excluded. Spending data were winsorized for the top and bottom 1% by radiotherapy technology type.

### Statistical Analysis

Patient characteristics were compared between those with TM and those with MA using standardized mean differences, with values greater than 0.10 indicating clinically important differences (Table 1). For each of the 3 outcomes, covariates included in the model were cancer type, age, dual-eligibility status, and patient comorbidities. For the first outcome, type of radiotherapy, we used logistic regression models for each technology type, adjusted for the covariates, to estimate differences in the proportion of episodes in which a specific type of technology was used. Odds ratios (ORs) were produced to compare technology types between TM and MA episodes. The second outcome, treatment length, was analyzed using a negative binomial model (suitable for count data, such as number of treatments), adjusted for the covariates, to determine differences in treatment length between TM and MA episodes overall and by type of technology. Rate ratios (RRs) were produced to compare treatment length between TM and MA episodes. The third outcome, 90-day radiotherapy spending, was analyzed using a log λ regression model (appropriate for positively skewed data, such as spending), adjusted for the covariates, to analyze differences in mean spending for all episodes and by type of technology. Rate ratios (RRs) were produced to compare spending between TM and MA episodes. To facilitate interpretation, estimated means from alternative linear regression models, adjusted for the covariates, were used across all 3 outcomes to assess differences between TM and MA episodes. Analyses were repeated by cancer type for the 3 most common cancer types. Statistical significance was determined as a 95% CI excluding 1 and an α < .05, with all tests conducted as 2-sided. All analyses were performed using SAS, version 9.4 (SAS Institute, Inc).^25^

We performed 2 sensitivity analyses. First, we repeated the aforementioned analyses using all MA episodes from all MA plans instead of solely from highly reliable MA plans (eTable 3 in Supplement 1). Second, the analyses for each of the 3 outcomes were performed accounting for county fixed effects and radiotherapy center type in addition to age, dual-eligibility status, comorbidities, and cancer type (eTable 4 in Supplement 1).

## Results

### Study Cohort

The cohort included a total of 31 563 radiotherapy episodes (30 941 patients), with 22 594 TM episodes (71.58%; 22 124 patients [71.50%]) and 8969 MA episodes (28.42%; 8817 patients [28.50%]). Among those with TM, mean (SD) age was 74.76 [6.57] years; 49.24% of episodes were in females, and 50.76% were in males Among those with MA, mean (SD) age was 74.51 [6.24] years; 48.22% of episodes were in females, and 51.78% were in males. In the overall patient cohort, those aged 65 to 74 years accounted for 17 220 episodes (54.56%). Female and male patients accounted for 15 450 (48.95%) and 16 113 (51.05%) episodes, respectively. Dual-eligible and non–dual-eligible patients accounted for 3849 (12.19%) and 27 714 (87.81%) episodes, respectively. African American or Black patients accounted for 2902 episodes (9.19%); Hispanic or Latinx patients, 1603 (5.08%); White patients, 25 619 (81.17%); patients reporting other race and ethnicity, 953 (3.02%); and patients with unknown race and ethnicity, 486 (1.54%). Breast, lung, and prostate cancer (the 3 most common cancer types in our study) accounted for 6008 (19.03%), 5649 (17.90%), and 6153 (19.49%) episodes, respectively. Freestanding radiotherapy centers accounted for 12 069 episodes (38.24%). Patients died during the treatment year in 6372 of all episodes (20.19%). The most common comorbidities were chronic obstructive pulmonary disease in 8206 episodes (26.00%), diabetes in 8103 (25.67%), heart arrhythmia in 5887 (18.65%), vascular disease in 6937 (21.98%), and congestive heart failure in 4742 (15.02%). Table 1 and eTable 1 in Supplement 1 contain baseline demographic variables for each episode, stratified by TM and MA episodes.

### Type of Radiotherapy Technology, Treatment Length, and Spending for All Cancers

**Figure 1.**
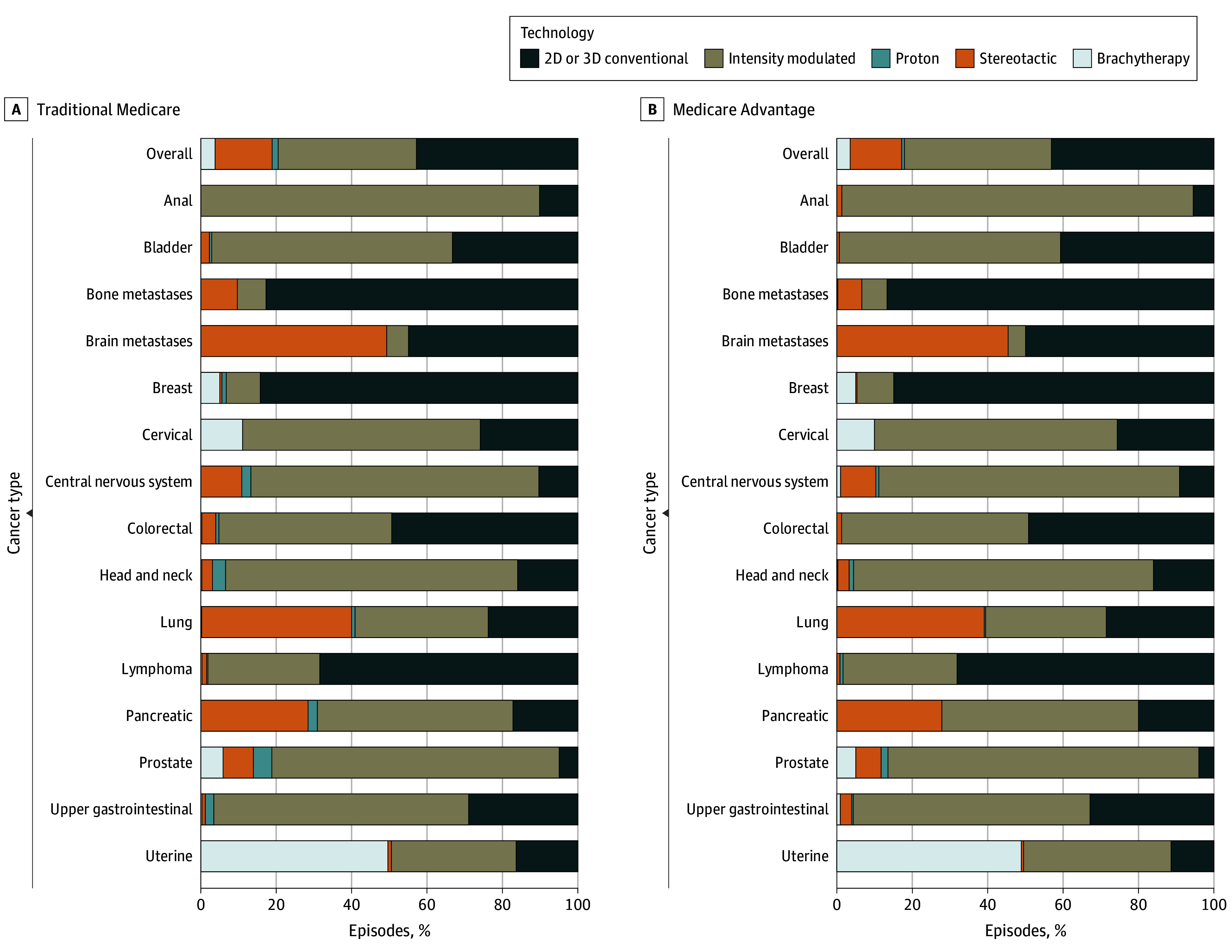
Use of Radiotherapy Technology by Cancer Type Across 90-Day Radiotherapy Episodes Covered by Medicare Advantage vs Traditional Medicare in 2018 2D indicates 2-dimensional; 3D, 3-dimensional.

After adjusting for cancer type, age, dual-eligibility status, and patient comorbidities, for MA episodes, we found higher odds of 2D-3D radiotherapy use (3962 [44.17% (95% CI, 43.39%-44.96%)] vs 9584 [42.43% (95% CI, 41.93%-42.92%)]; OR, 1.13 [95% CI, 1.06-1.21]) compared with TM episodes, but there was no difference in odds of IMRT use (3390 [37.80% (95% CI, 37.00%-38.60%)] vs 8346 [36.94% (95% CI, 36.44%-37.44%)]; OR, 1.06 [95% CI, 0.99-1.13]). Compared with TM episodes, for MA episodes, we found lower odds of proton therapy use (52 [0.58% (95% CI, 0.34%-0.82%)] vs 373 [1.65% (95% CI, 1.50%-1.80%)]; OR, 0.36 [95% CI, 0.27-0.48]) and lower odds of stereotactic radiotherapy use (n = 1235 [13.77% (95% CI, 13.13%-14.41%)] vs 3391 [15.01% (95% CI, 14.61%-15.41%)]; OR, 0.87 [95% CI, 0.81-0.95]). Brachytherapy use was similar in MA and TM episodes (318 [3.55% (95% CI, 3.20%-3.90%)] vs 859 [3.80% (95% CI, 3.58%-4.02%)]; OR, 0.91 [95% CI, 0.79-1.06]). Furthermore, 52 episodes (0.16%) had an equal number of treatments for the top 2 technology types and were excluded from the analysis. Figure 1 and eFigures 1 to 16 in Supplement 1 show the adjusted proportion of episodes in which each type of radiotherapy technology was used, stratified by insurance type, overall and by cancer type.

**Figure 2.**
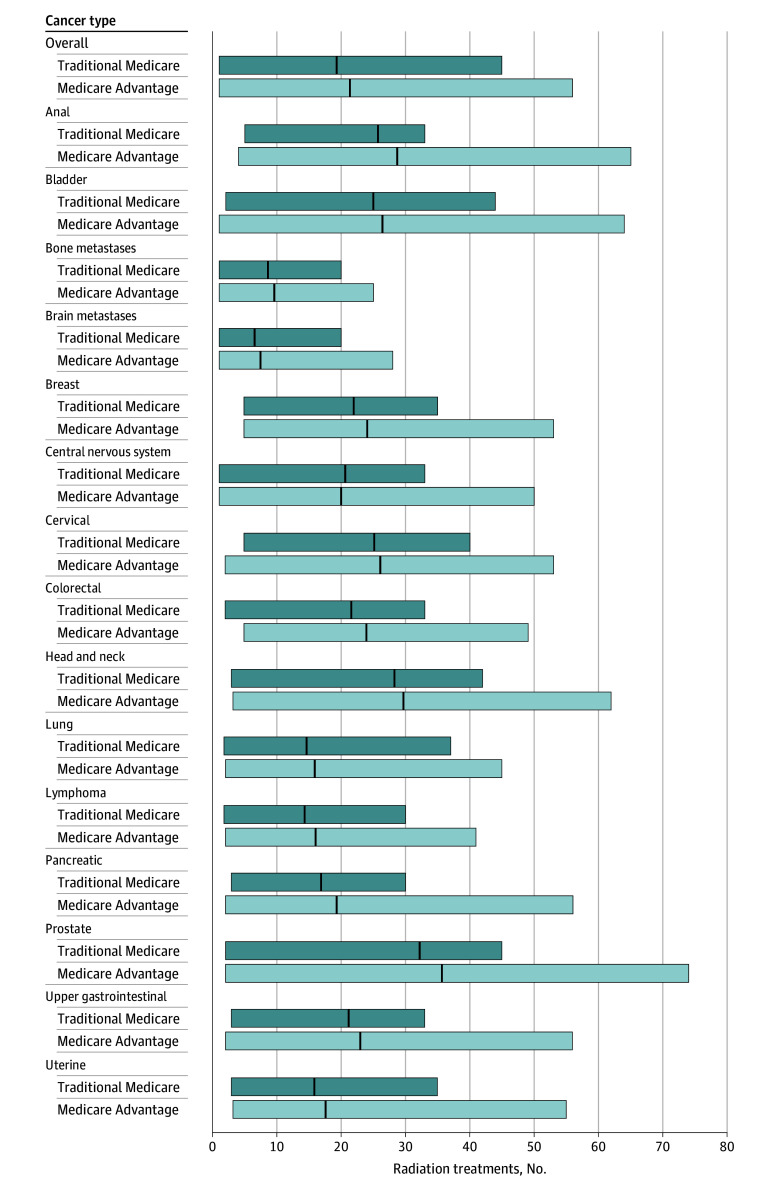
Mean Treatment Length Per Episode Across 90-Day Radiotherapy Episodes Covered by Medicare Advantage vs Traditional Medicare in 2018 Vertical lines within bars represent the mean number of radiotherapy treatments per episode, calculated as the number of radiotherapy treatment delivery codes, and outer edges of bars, 2.5th and 97.5th percentiles.

Overall, the adjusted mean treatment length per episode was significantly greater for MA episodes than for TM episodes (21.38 [95% CI, 21.14-21.61] vs 19.48 [95% CI, 19.33-19.62] treatments; RR, 1.10 [95% CI, 1.08-1.11]). Treatment length was significantly greater for MA episodes than for TM episodes for 2D-3D radiotherapy (17.32 [95% CI, 17.07-17.57] vs 16.00 [95% CI, 15.84-16.16] treatments; RR, 1.08 [95% CI, 1.06-1.10]), IMRT (33.23 [95% CI, 32.90-33.57] vs 30.61 [95% CI, 30.39-30.83] treatments; RR, 1.09 [95% CI, 1.07-1.10]), proton therapy (36.88 [95% CI, 34.49-39.28] treatments vs 31.87 [95% CI, 30.95-32.80] treatments; RR, 1.15 [95% CI, 1.05-1.26]), stereotactic radiotherapy (4.28 [95% CI, 4.19-4.38] vs 3.94 [95% CI, 3.88-4.00] treatments; RR, 1.09 [95% CI, 1.05-1.12]), and brachytherapy (7.57 [95% CI, 7.19-7.96] vs 7.18 [95% CI, 6.94-7.42] treatments; RR, 1.06 [95% CI, 1.00-1.13]). Figure 2 shows the adjusted mean number of treatments per episode, stratified by insurance type, overall and by cancer type.

**Figure 3.**
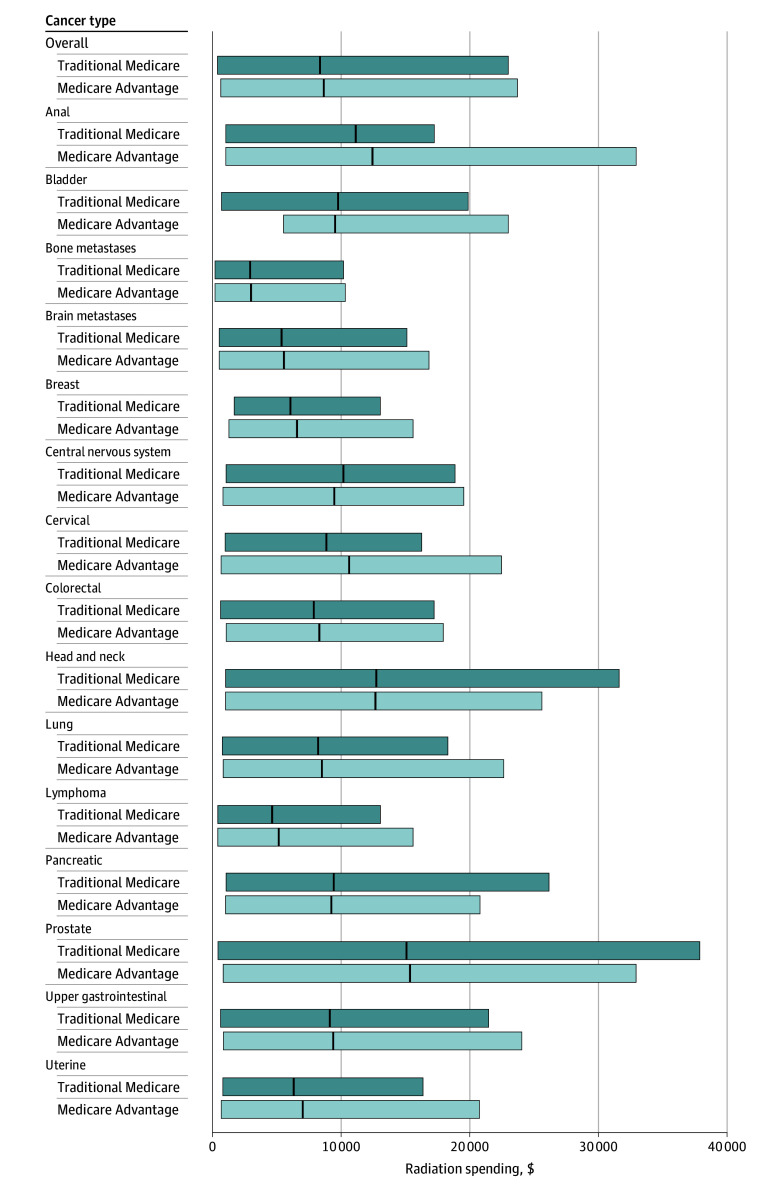
Mean Estimated Spending Per Episode Across 90-Day Radiotherapy Episodes Covered by Medicare Advantage vs Traditional Medicare in 2018 Vertical lines within bars represent mean estimated spending per episode and outer edges of bars, 2.5th and 97.5th percentiles.

Overall estimated radiotherapy spending per episode was significantly higher for MA episodes than for TM episodes ($8677.56 [95% CI, $8566.58-$8788.54] vs $8393.20 [95% CI, $8323.34-$8463.05]; RR, 1.04 [95% CI, 1.02-1.06]). Radiotherapy spending was also significantly higher for MA episodes for 2D-3D radiotherapy ($4302.74 [95% CI, $4230.25-$4375.23] vs $3960.26 [95% CI, $3914.35-$4006.17]; RR, 1.08 [95% CI, 1.06-1.11]), IMRT ($13 933.05 [95% CI, $13 770.87-$14 095.23] vs $13 380.61 [95% CI, $13 275.62-$13 485.60]; RR, 1.04 [95% CI, 1.02-1.06]), proton therapy ($36 168.64 [95% CI, $33 673.62-$38 663.65] vs $31 691.08 [95% CI, $30 723.04-$32 659.12]; RR, 1.14 [95% CI, 1.02-1.27]), stereotactic radiotherapy ($8166.82 [95% CI, $8001.84-$8331.80] vs $7512.28 [95% CI, $7414.33-$7610.23]; RR, 1.09 [95% CI, 1.06-1.12]), and brachytherapy ($3765.26 [95% CI, $3580.83-$3949.68] vs $3205.83 [95% CI, $3090.97-$3320.69]; RR, 1.29 [95% CI, 1.19-1.39]). Overall results for the 3 outcomes of interest can be found in Table 2. Figure 3 shows estimated radiotherapy spending per episode, stratified by insurance type, overall and by cancer type.

**Table 2.** Regression Results Adjusted for Cancer Type, Age, Dual-Eligibility Status, and Patient Comorbidities

Radiotherapy technology	Episodes, No. (% [95% CI])^a^		OR (95% CI)^b^	*P* value	Treatments, No. (95% CI)^a^		RR (95% CI)^c^	*P* value	Spending, $ (95% CI)^a^		RR (95%CI)^d^	*P* value
	MA	TM			MA	TM			MA	TM		
Overall	NA	NA	NA	NA	21.38 (21.14-21.61)	19.48 (19.33-19.62)	1.10 (1.08-1.11)	<.001	8677.56 (8566.58-8788.54)	8393.20 (8323.34-8463.05)	1.04 (1.02-1.06)	<.001
2D or 3D conventional	3962 (44.17 [43.39-44.96])	9584 (42.43 [41.93-42.92])	1.13 (1.06-1.21)	<.001	17.32 (17.07-17.57)	16.00 (15.84-16.16)	1.08 (1.06-1.10)	<.001	4302.74 (4230.25-4375.23)	3960.26 (3914.35-4006.17)	1.08 (1.06-1.11)	<.001
IMRT	3390 (37.80 [37.00-38.60])	8346 (36.94 [36.44-37.44])	1.06 (0.99-1.13)	.08	33.23 (32.90-33.57)	30.61 (30.39-30.83)	1.09 (1.07-1.10)	<.001	13 933.05 (13 770.87-14 095.23)	13 380.61 (13 275.62-13 485.60)	1.04 (1.02-1.06)	<.001
Proton	52 (0.58 [0.34-0.82])	373 (1.65 [1.50-1.80])	0.36 (0.27-0.48)	<.001	36.88 (34.49-39.28)	31.87 (30.95-32.80)	1.15 (1.05-1.26)	.002	36 168.64 (33 673.62-38 663.65)	31 691.08 (30 723.04-32 659.12)	1.14 (1.02-1.27)	.02
Stereotactic	1235 (13.77 [13.13-14.41])	3391 (15.01 [14.61-15.41])	0.87 (0.81-0.95)	.001	4.28 (4.19-4.38)	3.94 (3.88-4.00)	1.09 (1.05-1.12)	<.001	8166.82 (8001.84-8331.80)	7512.28 (7414.33-7610.23)	1.09 (1.06-1.12)	<.001
Brachytherapy	318 (3.55 [3.20-3.90])	859 (3.80 [3.58-4.02])	0.91 (0.79-1.06)	.23	7.57 (7.19-7.96)	7.18 (6.94-7.42)	1.06 (1.00-1.13)	.045	3765.26 (3580.83-3949.68)	3205.83 (3090.97-3320.69)	1.29 (1.19-1.39)	<.001

Abbreviations: 2D, 2-dimensional; 3D, 3-dimensional; IMRT, intensity-modulated radiotherapy; MA, Medicare Advantage; NA, not applicable; OR, odds ratio; RR, rate ratio; TM, traditional Medicare.

^a^ Data are presented as estimated means from alternative linear regression models. Covariates included insurance type (TM vs MA), cancer type, age, dual-eligibility status, and comorbidities.

^b^ From logistic regression.

^c^ From a negative binomial model.

^d^ From a log λ regression model.

When comparing the results of linear regression with results of other types of regression, including a logistic regression model for technology type, a negative binomial model for estimated treatment length, and a log λ model for estimated spending, the results were qualitatively similar for all 3 outcomes of interest. When comparing mean treatment length calculated using all types of technology with mean treatment length calculated using only the majority type of technology for each category (grouped by majority type of radiotherapy technology), the mean treatment length differed by less than 1.2% (95% CI, 0.7%-1.6%), or 0.4 treatments (95% CI, 0.2-0.5 treatments), for TM and by 1.4% (95% CI, 1.2%-1.6%) or less (0.5 [95% CI, 0.4-0.6] treatments) for MA. eTable 5 in Supplement 1 shows the mean treatment length calculated using all types of technology and the mean treatment length calculated using the majority type of technology only, grouped by majority technology type and insurance type.

### Type of Radiotherapy Technology, Treatment Length, and Spending for Breast, Lung, and Prostate Cancers

Compared with TM episodes, for MA episodes, we found lower adjusted rates and odds of proton therapy use for breast (<12 [0.22% (95% CI, 0.00%-0.64%)] vs 44 [1.03% (95% CI, 0.77%-1.30%)]; OR, 0.22 [95% CI, 0.08-0.61]) and prostate (36 [1.88% (95% CI, 1.02%-2.75%)] vs 204 [4.82% (95% CI, 4.24%-5.40%)]; OR, 0.38 [95% CI, 0.26-0.54]) cancer treatment, but there was no difference in adjusted rates and odds of use for lung cancer treatment (<12 [0.45% (95% CI, 0.02%-0.89%)] vs 37 [0.90% (95% CI, 0.64%-1.18%)]; OR, 0.48 [95% CI, 0.22-1.09]). The mean treatment length per episode was greater for MA than for TM episodes for breast (24.08 [95% CI, 23.66-24.50] vs 21.92 [95% CI, 21.65-22.19] treatments; RR, 1.10 [95% CI, 1.07-1.12]), prostate (35.63 [95% CI, 34.93-36.34] vs 32.19 [95% CI, 31.71-32.66]; RR, 1.10 [95% CI, 1.07-1.14]), and lung (15.85 [95% CI, 15.24-16.46] vs 14.61 [95% CI, 14.23-14.99] treatments; RR, 1.08 [95% CI, 1.03-1.13]) cancer treatment. Radiotherapy spending per episode was higher for MA episodes than for TM episodes for breast cancer ($6590.09 [95% CI, $6426.85-$6753.34] vs $6091.26 [95% CI, $5987.44-$6195.09]; RR, 1.08 [95% CI, 1.05-1.11]), but there was no difference for prostate ($15 369.20 [95% CI, $15 011.50-$15 726.90] vs $15 087.33 [95% CI, $14 846.65-$15 328.00]; RR, 1.02 [95% CI, 0.98-1.06]) or lung ($8512.79 [95% CI, $8256.81-$8768.78] vs $8224.59 [95% CI, $8065.18-$8383.99]; RR, 1.03 [95% CI, 0.99-1.08]) cancer treatment. The outcomes for the remaining cancer types are shown in Figure 1, Figure 2, and Figure 3 and eFigures 1 to 16 in Supplement 1.

### Sensitivity Analyses

For both sensitivity analyses, results were similar to those of our primary analysis. Data are provided in eTables 3 to 5 in Supplement 1.

## Discussion

We analyzed radiotherapy utilization and spending patterns for patients with cancer enrolled in MA vs TM. We found that for radiotherapy episodes covered by MA, there were higher odds of less-advanced, 2D-3D therapy use and lower odds of advanced stereotactic radiotherapy and proton beam therapy use compared with TM episodes. Furthermore, MA episodes had longer mean treatment length overall and for the 3 most common solid cancers (breast, lung, and prostate) and higher estimated radiotherapy spending for breast cancer. We conjecture that decreased utilization of advanced technologies may be due to prior authorization and limited networks under MA.

Our study found that MA radiotherapy episodes were less likely to include use of more advanced technologies, such as proton therapy and stereotactic radiotherapy. Several factors may contribute to the decreased use of proton therapy. First, MA plans, unlike TM plans, may use prior authorization to limit use of more costly therapies.^16,17,18^ Second, proton therapy is typically offered at large tertiary academic cancer centers, and MA networks might favor contracting with smaller health systems to contain costs.^26^ Additionally, research suggests that use of proton therapy has enhanced value only for certain cancer types and patient subsets, meaning efforts to curb its use may reflect appropriate cost-saving measures.^27,28^ However, as proton therapy remains an area of active investigation for other disease sites, with new studies further refining and expanding the role of proton therapy since 2018,^29,30,31^ it is crucial to ensure that prior authorization does not restrict access when use of such technologies is evidence based. Our results align with previous studies indicating lower initial approval rates for proton therapy under private insurance compared with TM.^16,17^ Another study showed an increase in prior authorization requests for IMRT, which was associated with treatment delays.^32^ Therefore, our results showing no difference in IMRT utilization between TM and MA might suggest that while prior authorization does not decrease its use, it may introduce treatment delays. Consistent with previous studies, we found that racial and ethnic minority patients were more likely to enroll in MA; hence, any approval delays could exacerbate inequalities.^32,33^

Our study found that the mean treatment length was significantly longer for MA episodes compared with TM episodes across all radiotherapy technologies and the 3 most common cancer types. This aligns with our finding that in MA episodes, stereotactic radiotherapy, which may require fewer treatments, was less likely to be used. For example, stereotactic radiotherapy episodes typically involve 1 to 5 treatments, whereas conventional radiotherapy, such as 2D-3D radiotherapy or IMRT, extends over many weeks. Courses with a higher number of treatments may exceed American Society for Radiation Oncology recommendations^34^ and pose additional time and transportation barriers for patients.^35^ Additionally, the mean estimated radiotherapy spending per episode was significantly higher for MA episodes than for TM episodes overall, across all radiotherapy technologies, and for breast cancer. A recent study showed that MA patients had lower chemotherapy and immunotherapy utilization and spending, with similar survival outcomes, compared with TM patients.^36^ Thus, these conflicting studies suggest that neither TM nor MA consistently delivers better value for oncology patients.^36^ Ultimately, whether MA meets the value proposition for individual patients depends on personal priorities, such as balancing lower premiums and out-of-pocket costs against preauthorization requirements and potentially limited networks.^7,8,9,10,11^ Limited networks may have greater importance for patients undergoing radiotherapy given the travel burden to and from daily treatments. It is crucial to ensure that older adults fully understand these tradeoffs when enrolling in either TM or MA.^37^

### Limitations

Our study has several limitations. First, due to unavailable MA reimbursement data, we standardized MA spending using the TM Physician Fee Schedule, a method supported by prior studies due to high correlation between TM and MA reimbursement.^38,39^ We focused on radiotherapy spending, not overall patient spending, to ensure methodologic clarity and internal validity. Second, claims data lack granular clinical information (ie, time from diagnosis to radiotherapy initiation) and insurance approval rates for radiation therapy, a growing area of concern with MA plans, both of which necessitate further evaluation.^10,40,41^ Prior authorization and payment denials impact care delivery; a study found that 13% of proton therapy episodes under MA were denied, even after appeals.^17^ Furthermore, MA plans may use strategies that favor selecting healthier patients through patient incentives and favorable payment adjustments by county, aiming to increase returns through favorable selection.^42,43^ We addressed potential selection effects through our sensitivity analysis, which adjusted for county effects and comorbidities. Third, differences in data reliability between MA plans present another limitation, representing potential sources of bias of unknown directionality. However, the magnitude of this bias was likely minimal, as results remained consistent when including all MA plans and only those with high-quality data. We used a validated approach to identify highly reliable MA plans, acknowledging that this reduces the sample size.^23,41^ Improved MA data completeness, through improved automation and standardization of reporting quality metrics, could mitigate biases and benefit researchers and policymakers. Fourth, as a cross-sectional study, our study could not assess temporal trends, necessitating further longitudinal studies. Fifth, by categorizing episodes by majority modality, some mixed treatments, such as combined brachytherapy and external radiotherapy, would likely be classified as external radiotherapy. Further nuanced studies by mixed technology groups should be explored.

## Conclusions

In this cross-sectional study, we examined radiotherapy utilization and spending among patients with cancer undergoing radiotherapy episodes covered by MA and TM. We found that for MA radiotherapy episodes, there was higher estimated spending and greater mean treatment length than for TM radiotherapy episodes. Although among MA episodes, there was lower utilization of more expensive, advanced treatment modalities, MA was not associated with cost savings compared with TM. Additionally, our study found that patients covered by MA had higher mean treatment length than those covered by TM, which may represent increased transportation challenges for these patients. Whether MA meets the value proposition for radiation oncology requires further investigation.

## Notes

Supplement 1. **eTable 1.** Patient Comorbidities Included in Sensitivity Analyses Listed by Hierarchical Condition Category **eTable 2.** *ICD-10* Codes Used to Define Each of the 15 Cancer Types of Interest **eTable 3.** Regression Results for all Medicare Advantage Plans, Adjusted for Cancer Type, Age, Dual-Eligibility Status, and Patient Comorbidities **eTable 4.** Regression Results Adjusted for Cancer Type, Age, Dual-Eligibility Status, Patient Comorbidities, County, and RT Center Type **eTable 5.** Mean Unadjusted Number of Treatments for All Modalities and Majority Modality Only **eFigure 1.** Radiotherapy Technology Type Utilization Across 90-Day Radiation Therapy Episodes Overall, MA vs TM, 2018 **eFigure 2.** Radiotherapy Technology Type Utilization Across 90-Day Radiation Therapy Episodes for Anal Cancer, MA vs TM, 2018 **eFigure 3.** Radiotherapy Technology Type Utilization Across 90-Day Radiation Therapy Episodes for Bladder Cancer, MA vs TM, 2018 **eFigure 4.** Radiotherapy Technology Type Utilization Across 90-Day Radiation Therapy Episodes for Bone Metastases, MA vs TM, 2018 **eFigure 5.** Radiotherapy Technology Type Utilization Across 90-Day Radiation Therapy Episodes for Brain Metastases, MA vs TM, 2018 **eFigure 6.** Radiotherapy Technology Type Utilization Across 90-Day Radiation Therapy Episodes for Breast Cancer, MA vs TM, 2018 **eFigure 7.** Radiotherapy Technology Type Utilization Across 90-Day Radiation Therapy Episodes for Cervical Cancer, MA vs TM, 2018 **eFigure 8.** Radiotherapy Technology Type Utilization Across 90-Day Radiation Therapy Episodes for CNS Cancer, MA vs TM, 2018 **eFigure 9.** Radiotherapy Technology Type Utilization Across 90-Day Radiation Therapy Episodes for Colorectal Cancer, MA vs TM, 2018 **eFigure 10.** Radiotherapy Technology Type Utilization Across 90-Day Radiation Therapy Episodes for Head and Neck Cancer, MA vs TM, 2018 **eFigure 11.** Radiotherapy Technology Type Utilization Across 90-Day Radiation Therapy Episodes for Lung Cancer, MA vs TM, 2018 **eFigure 12.** Radiotherapy Technology Type Utilization Across 90-Day Radiation Therapy Episodes for Lymphoma, MA vs TM, 2018 **eFigure 13.** Radiotherapy Technology Type Utilization Across 90-Day Radiation Therapy Episodes for Pancreatic Cancer, MA vs TM, 2018 **eFigure 14.** Radiotherapy Technology Type Utilization Across 90-Day Radiation Therapy Episodes for Prostate Cancer, MA vs TM, 2018 **eFigure 15.** Radiotherapy Technology Type Utilization Across 90-Day Radiation Therapy Episodes for Upper Gastrointestinal Cancer, MA vs TM, 2018 **eFigure 16.** Radiotherapy Technology Type Utilization Across 90-Day Radiation Therapy Episodes for Uterine Cancer, MA vs TM, 2018

Supplement 2.
Data Sharing Statement
