# Supplementary material for: Radiotherapy Utilization in Traditional Medicare and Medicare Advantage
Source: JAMA Netw Open. 2025 Apr 2;8(4):e253018. doi: 10.1001/jamanetworkopen.2025.3018 (PMC11966333; doi:10.1001/jamanetworkopen.2025.3018)
Supplement: Supplement 1. — eTable 1. Patient Comorbidities Included in Sensitivity Analyses Listed by Hierarchical Condition Category eTable 2. ICD-10 Codes Used to Define Each of the 15 Cancer Types of Interest eTable 3. Regression Results for all Medicare Advantage Plans, Adjusted for Cancer Type, Age, Dual-Eligibility Status, and Patient Comorbidities eTable 4. Regression Results Adjusted for Cancer Type, Age, Dual-Eligibility Status, Patient Comorbidities, County, and RT Center Type eTable 5. Mean Unadjusted Number of Treatments for All Modalities and Majority Modality Only eFigure 1. Radiotherapy Technology Type Utilization Across 90-Day Radiation Therapy Episodes Overall, MA vs TM, 2018 eFigure 2. Radiotherapy Technology Type Utilization Across 90-Day Radiation Therapy Episodes for Anal Cancer, MA vs TM, 2018 eFigure 3. Radiotherapy Technology Type Utilization Across 90-Day Radiation Therapy Episodes for Bladder Cancer, MA vs TM, 2018 eFigure 4. Radiotherapy Technology Type Utilization Across 90-Day Radiation Therapy Episodes for Bone Metastases, MA vs TM, 2018 eFigure 5. Radiotherapy Technology Type Utilization Across 90-Day Radiation Therapy Episodes for Brain Metastases, MA vs TM, 2018 eFigure 6. Radiotherapy Technology Type Utilization Across 90-Day Radiation Therapy Episodes for Breast Cancer, MA vs TM, 2018 eFigure 7. Radiotherapy Technology Type Utilization Across 90-Day Radiation Therapy Episodes for Cervical Cancer, MA vs TM, 2018 eFigure 8. Radiotherapy Technology Type Utilization Across 90-Day Radiation Therapy Episodes for CNS Cancer, MA vs TM, 2018 eFigure 9. Radiotherapy Technology Type Utilization Across 90-Day Radiation Therapy Episodes for Colorectal Cancer, MA vs TM, 2018 eFigure 10. Radiotherapy Technology Type Utilization Across 90-Day Radiation Therapy Episodes for Head and Neck Cancer, MA vs TM, 2018 eFigure 11. Radiotherapy Technology Type Utilization Across 90-Day Radiation Therapy Episodes for Lung Cancer, MA vs TM, 2018 eFigure 12. Radiotherapy Technology Type [file jamanetwopen-e253018-s001.pdf]

Supplementary Online Content

Hogan J, Orav EJ, Zhang T, et al. Radiotherapy utilization in traditional Medicare and Medicare Advantage. *JAMA Netw Open*. 2025;8(4):e253018. doi:10.1001/jamanetworkopen.2025.3018

eTable 1. Patient Comorbidities Included in Sensitivity Analyses Listed by Hierarchical Condition Category

eTable 2. *ICD-10* Codes Used to Define Each of the 15 Cancer Types of Interest

eTable 3. Regression Results for all Medicare Advantage Plans, Adjusted for Cancer Type, Age, Dual-Eligibility Status, and Patient Comorbidities

eTable 4. Regression Results Adjusted for Cancer Type, Age, Dual-Eligibility Status, Patient Comorbidities, County, and RT Center Type

eTable 5. Mean Unadjusted Number of Treatments for All Modalities and Majority Modality Only

eFigure 1. Radiotherapy Technology Type Utilization Across 90-Day Radiation Therapy Episodes Overall, MA vs TM, 2018

eFigure 2. Radiotherapy Technology Type Utilization Across 90-Day Radiation Therapy Episodes for Anal Cancer, MA vs TM, 2018

eFigure 3. Radiotherapy Technology Type Utilization Across 90-Day Radiation Therapy Episodes for Bladder Cancer, MA vs TM, 2018

eFigure 4. Radiotherapy Technology Type Utilization Across 90-Day Radiation Therapy Episodes for Bone Metastases, MA vs TM, 2018

eFigure 5. Radiotherapy Technology Type Utilization Across 90-Day Radiation Therapy Episodes for Brain Metastases, MA vs TM, 2018

eFigure 6. Radiotherapy Technology Type Utilization Across 90-Day Radiation Therapy Episodes for Breast Cancer, MA vs TM, 2018

eFigure 7. Radiotherapy Technology Type Utilization Across 90-Day Radiation Therapy Episodes for Cervical Cancer, MA vs TM, 2018

eFigure 8. Radiotherapy Technology Type Utilization Across 90-Day Radiation Therapy Episodes for CNS Cancer, MA vs TM, 2018

eFigure 9. Radiotherapy Technology Type Utilization Across 90-Day Radiation Therapy Episodes for Colorectal Cancer, MA vs TM, 2018

eFigure 10. Radiotherapy Technology Type Utilization Across 90-Day Radiation Therapy Episodes for Head and Neck Cancer, MA vs TM, 2018

eFigure 11. Radiotherapy Technology Type Utilization Across 90-Day Radiation Therapy Episodes for Lung Cancer, MA vs TM, 2018

eFigure 12. Radiotherapy Technology Type Utilization Across 90-Day Radiation Therapy Episodes for Lymphoma, MA vs TM, 2018

© 2025 Hogan J et al. *JAMA Network Open*.

eFigure 13. Radiotherapy Technology Type Utilization Across 90-Day Radiation Therapy Episodes for Pancreatic Cancer, MA vs TM, 2018

eFigure 14. Radiotherapy Technology Type Utilization Across 90-Day Radiation Therapy Episodes for Prostate Cancer, MA vs TM, 2018

eFigure 15. Radiotherapy Technology Type Utilization Across 90-Day Radiation Therapy Episodes for Upper Gastrointestinal Cancer, MA vs TM, 2018

eFigure 16. Radiotherapy Technology Type Utilization Across 90-Day Radiation Therapy Episodes for Uterine Cancer, MA vs TM, 2018

This supplementary material has been provided by the authors to give readers additional information about their work.

© 2025 Hogan J et al. *JAMA Network Open*.

eTable 1. Patient comorbidities included in sensitivity analyses listed by Hierarchical Condition Category (HCC)

Characteristics	Category	N Episodes TM	N Episodes MA	SMD
Overall		22,594	8969	
HCC1	HIV/AIDS	39 (0.17%)	19 (0.21%)	-0.0092
HCC2	Septicemia, Sepsis, Systemic Inflammatory Response Syndrome/Shock	2191 (9.70%)	858 (9.57%)	0.0044
HCC6	Opportunistic Infections	238 (1.05%)	97 (1.08%)	-0.0027
HCC8	Metastatic Cancer and Acute Leukemia	8885 (39.32%)	3220 (35.90%)	0.0704
HCC9	Lung and Other Severe Cancers	2994 (13.25%)	1149 (12.81%)	0.0130
HCC10	Lymphoma and Other Cancers	1663 (7.36%)	617 (6.88%)	0.0186
HCC11	Colorectal, Bladder, and Other Cancers	1317 (5.83%)	561 (6.25%)	-0.0180
HCC12	Breast, Prostate, and Other Cancers and Tumors	6255 (27.68%)	2567 (28.62%)	-0.0209
HCC21	Protein-Calorie Malnutrition	2922 (12.93%)	1126 (12.55%)	0.0113
HCC22	Morbid Obesity	1015 (4.49%)	366 (4.08%)	0.0201
HCC23	Other Significant Endocrine and Metabolic Disorders	1760 (7.79%)	630 (7.02%)	0.0289
HCC27	End-Stage Liver Disease	185 (0.82%)	61 (0.68%)	0.0158
HCC28	Cirrhosis of Liver	178 (0.79%)	66 (0.74%)	0.0059
HCC29	Chronic Hepatitis	97 (0.43%)	34 (0.38%)	0.0078
HCC33	Intestinal Obstruction/Perforation	810 (3.59%)	319 (3.56%)	0.0015
HCC34	Chronic Pancreatitis	83 (0.37%)	28 (0.31%)	0.0093
HCC35	Inflammatory Bowel Disease	210 (0.93%)	81 (0.90%)	0.0028
HCC39	Bone/Joint/Muscle Infections/Necrosis	250 (1.11%)	74 (0.83%)	0.0279
HCC40	Rheumatoid Arthritis and Inflammatory Connective Tissue Disease	1065 (4.71%)	383 (4.27%)	0.0212
HCC46	Severe Hematological Disorders	205 (0.91%)	77 (0.86%)	0.0052
HCC47	Disorders of Immunity	2764 (12.23%)	1056 (11.77%)	0.0141
HCC48	Coagulation Defects and Other Specified Hematological Disorders	2450 (10.84%)	876 (9.77%)	0.0351
HCC51	Dementia With Complications	NA	NA	NA
HCC52	Dementia Without Complication	NA	NA	NA
HCC54	Drug/Alcohol Psychosis	18 (0.08%)	NA	NA
HCC55	Drug/Alcohol Dependence	478 (2.12%)	208 (2.23%)	-0.0140
HCC57	Schizophrenia	62 (0.27%)	21 (0.23%)	0.0079
HCC58	Major Depressive, Bipolar, and Paranoid Disorders	612 (2.71%)	192 (2.14%)	0.0361
HCC70	Quadriplegia	37 (0.16%)	NA	NA
HCC71	Paraplegia	91 (0.40%)	38 (0.42%)	-0.0033
HCC72	Spinal Cord Disorders/Injuries	455 (2.01%)	156 (1.74%)	0.0199
HCC73	Amyotrophic Lateral Sclerosis and Other Motor Neuron Disease	NA	NA	NA
HCC74	Cerebral Palsy	14 (0.06%)	NA	NA

© 2025 Hogan J et al. *JAMA Network Open*.

HCC75	Polyneuropathy	602 (2.66%)	187 (2.08%)	0.0371
HCC76	Muscular Dystrophy	NA	NA	NA
HCC77	Multiple Sclerosis	67 (0.30%)	19 (0.21%)	0.0162
HCC78	Parkinson's and Huntington's Diseases	187 (0.83%)	76 (0.85%)	-0.0022
HCC79	Seizure Disorders and Convulsions	958 (4.24%)	332 (3.70%)	0.0272
HCC80	Coma, Brain Compression/Anoxic Damage	1508 (6.67%)	526 (5.86%)	0.0330
HCC82	Respirator Dependence/Tracheostomy Status	196 (0.87%)	87 (0.97%)	-0.0109
HCC83	Respiratory Arrest	14 (0.06%)	NA	NA
HCC84	Cardio-Respiratory Failure and Shock	2834 (12.54%)	1133 (12.63%)	-0.0027
HCC86	Acute Myocardial Infarction	656 (2.90%)	207 (2.31%)	0.0365
HCC87	Unstable Angina and Other Acute Ischemic Heart Disease	368 (1.63%)	136 (1.52%)	0.0090
HCC88	Angina Pectoris	334 (1.48%)	127 (1.42%)	0.0052
HCC99	Cerebral Hemorrhage	389 (1.72%)	146 (1.63%)	0.0073
HCC100	Ischemic or Unspecified Stroke	547 (2.42%)	204 (2.27%)	0.0096
HCC103	Hemiplegia/Hemiparesis	685 (3.03%)	221 (2.46%)	0.0340
HCC104	Monoplegia, Other Paralytic Syndromes	95 (0.42%)	20 (0.22%)	0.0328
HCC106	Atherosclerosis of the Extremities with Ulceration or Gangrene	107 (0.47%)	38 (0.42%)	0.0074
HCC110	Cystic Fibrosis	NA	NA	NA
HCC112	Fibrosis of Lung and Other Chronic Lung Disorders	525 (2.32%)	140 (1.56%)	0.0531
HCC114	Aspiration and Specified Bacterial Pneumonias	826 (3.66%)	337 (3.76%)	-0.0054
HCC115	Pneumococcal Pneumonia, Empyema, Lung Abscess	456 (2.02%)	188 (2.10%)	-0.0055
HCC122	Proliferative Diabetic Retinopathy and Vitreous Hemorrhage	19 (0.08%)	NA	NA
HCC124	Exudative Macular Degeneration	40 (0.18%)	NA	NA
HCC134	Dialysis Status	165 (0.73%)	61(0.68%)	0.0059
HCC135	Acute Renal Failure	2834 (12.54%)	1176 (13.11%)	-0.0171
HCC136	Chronic Kidney Disease, Stage 5	33 (0.15%)	14 (0.16%)	-0.0026
HCC137	Chronic Kidney Disease, Severe (Stage 4)	118 (0.52%)	46 (0.51%)	0.0013
HCC138	Chronic Kidney Disease, Moderate (Stage 3)	NA	NA	NA
HCC139	Chronic Kidney Disease, Mild or Unspecified (Stages 1-2 or Unspecified)	NA	NA	NA
HCC140	Unspecified Renal Failure	NA	NA	NA
HCC141	Nephritis	NA	NA	NA
HCC157	Pressure Ulcer of Skin with Necrosis Through to Muscle, Tendon, or Bone	43 (0.19%)	20 (0.22%)	-0.0073
HCC158	Pressure Ulcer of Skin with Full Thickness Skin Loss	185 (0.82%)	60 (0.67%)	0.0171

© 2025 Hogan J et al. *JAMA Network Open*.

**HCC159**	Pressure Ulcer of Skin with Partial Thickness Skin Loss	NA	NA	NA
**HCC160**	Pressure Pre-Ulcer Skin Changes or Unspecified Stage	NA	NA	NA
**HCC161**	Chronic Ulcer of Skin, Except Pressure	325 (1.44%)	76 (0.85%)	0.0528
**HCC162**	Severe Skin Burn or Condition	NA	NA	NA
**HCC166**	Severe Head Injury	NA	NA	NA
**HCC167**	Major Head Injury	168 (0.74%)	60 (0.67%)	0.0088
**HCC169**	Vertebral Fractures without Spinal Cord Injury	683 (3.02%)	257 (2.87%)	0.0093
**HCC170**	Hip Fracture/Dislocation	455 (2.01%)	159 (1.77%)	0.0175
**HCC173**	Traumatic Amputations and Complications	52 (0.23%)	16 (0.18%)	0.0112
**HCC176**	Complications of Specified Implanted Device or Graft	805 (3.56%)	305 (3.40%)	0.0088
**HCC186**	Major Organ Transplant or Replacement Status	123 (0.53%)	46 (0.51%)	0.0043
**HCC188**	Artificial Openings for Feeding or Elimination	1210 (5.36%)	471 (5.25%)	0.0046
**HCC189**	Amputation Status, Lower Limb/Amputation Complications	99 (0.44%)	44 (0.49%)	-0.0078

TM: Traditional Medicare, MA: Medicare Advantage, SMD: Standardized Mean Difference, HCC: Hierarchical Condition Category.
Values <12 were unable to reported for privacy reasons and are marked as NA.

© 2025 Hogan J et al. *JAMA Network Open*.

eTable 2. *ICD-10* codes used to define each of the 15 cancer types of interest

Cancer Type	ICD-10 Codes
**Anal**	C21.xx
**Bladder**	C67.xx
**Bone Metastases**	C79.5x
**Brain Metastases**	C79.3x
**Breast**	C50.xx, D05.xx
**Cervical**	C53.xx
**Central Nervous System**	C70.xx, C71.xx, C72.xx
**Colorectal**	C18.xx, C19.xx, C20.xx
**Head & Neck**	C00.xx, C01.xx, C02.xx, C03.xx, C04.xx, C05.xx, C06.xx, C07.xx, C08.xx, C09.xx, C10.xx, C11.xx, C12.xx, C13.xx, C14.xx, C30.xx, C31.xx, C32.xx, C76.0x
**Lung**	C33.xx, C34.xx, C39.xx, C45.xx
**Lymphoma**	C81.xx, C82.xx, C83.xx, C84.xx, C85.xx, C86.xx, C88.xx, C91.4x
**Pancreatic**	C25.xx
**Prostate**	C61.xx
**Upper Gastrointestinal**	C15.xx, C16.xx, C17.xx
**Uterine**	C54.xx, C55.xx

ICD: International Classification of Diseases.

© 2025 Hogan J et al. *JAMA Network Open*.

eTable 3. Regression results for all Medicare Advantage plans, adjusted for cancer type, age, dual-eligibility status, and patient comorbidities

	MA [95% CI] n=12,509	TM [95% CI] n=22,553	OR/RR [95% CI]	P value
**Type of Radiation Treatment Technology (% of Episodes)**				
**2D/3D Conventional**	n=5624<br>44.88% [44.22%, 45.55%]	n=9578<br>42.39% [41.89%, 42.88%]	1.19 [1.12, 1.26]	<0.001
**Intensity Modulated**	n=4720<br>37.66% [36.98%, 38.33%]	n=8353<br>36.97% [36.47%, 37.47%]	1.05 [0.99, 1.11]	0.12
**Proton**	n=69<br>0.55% [0.35%, 0.74%]	n=371<br>1.64% [1.50%, 1.78%]	0.33 [0.26, 0.43]	<0.001
**Stereotactic**	n=1659<br>13.24% [12.70%, 13.78%]	n=3385<br>14.98% [14.58%, 15.38%]	0.83 [0.77, 0.89]	<0.001
**Brachytherapy**	n=437<br>3.49% [3.19%, 3.78%]	n=868<br>3.84% [3.62%, 4.06%]	0.88 [0.77, 1.01]	0.067
**Radiation Treatment Length (Number of Treatments)**				
**Overall**	21.07 [20.87 ,21.26]	19.50 [19.35 ,19.64]	1.08 [1.06, 1.09]	<0.001
**2D/3D Conventional**	17.19 [16.98 ,17.41]	16.02 [15.86 ,16.19]	1.07 [1.05, 1.09]	<0.001
**Intensity Modulated**	32.53 [32.24 ,32.82]	30.67 [30.44 ,30.89]	1.06 [1.05, 1.08]	<0.001
**Proton**	35.42 [33.25 ,37.60]	31.79 [30.84 ,32.73]	1.11 [1.02, 1.21]	0.012
**Stereotactic**	4.23 [4.14 ,4.32]	3.93 [3.87 ,3.99]	1.08 [1.04, 1.11]	<0.001
**Brachytherapy**	7.35 [7.02 ,7.68]	7.11 [6.86 ,7.35]	1.04 [0.98, 1.10]	0.19
**Radiation Therapy Spending (Dollars)**				
**Overall**	$8408.44 [$8314.27, $8502.6]	$8395.20 [$8325.14, $8465.25]	1.02 [1.00, 1.03]	0.021
**2D/3D Conventional**	$4332.86 [$4270.38, $4395.3]	$3966.43 [$3919.18, $4013.69]	1.08 [1.01, 1.11]	<0.001
**Intensity Modulated**	$13,399.64 [$13,255.88, $13,543.41]	$13,398.30 [$13,288.79, $13,507.81]	1.02 [1.01, 1.04]	0.0041
**Proton**	$34,709.37 [$32,447.34, $36,971.41]	$31,604.74 [$30,623.23, $32,586.24]	1.10 [0.99, 1.21]	0.075
**Stereotactic**	$8022.37 [$7876.20, $8168.5]	$7508.55 [$7407.88, $7609.22]	1.07 [1.04, 1.10]	<0.001
**Brachytherapy**	$3660.11 [$3496.56, $3823.66]	$3152.78 [$3032.85, $3272.72]	1.27 [1.18, 1.37]	<0.001

MA: Medicare Advantage, TM: Traditional Medicare, 2D: two-dimensional, 3D: three-dimensional, OR: Odds Ratio, RR: Rate Ratio, CI: Confidence Interval. Columns MA and TM contain predictive means from alternative linear regression models. Columns OR/RR (95% CI) contains the odds ratio/rate ratio with 95% CI using logistic regression when estimating the proportion of episodes using each treatment technology, a negative binomial model when estimating treatment length, and a log gamma regression model when estimating spending. For this analysis, covariates included insurance type (TM vs MA), cancer type, age, dual-eligibility status, and patient comorbidities. These results were obtained using data from all MA plans.

© 2025 Hogan J et al. *JAMA Network Open*.

eTable 4. Regression results adjusted for cancer type, age, dual-eligibility status, patient comorbidities, county, RT center type

	MA No [95% CI] n=8957	TM [95% CI] n=22,553	OR/RR [95% CI]	P value
**Type of Radiation Treatment Technology (% of Episodes)**				
**2D/3D Conventional**	n=3998<br>44.58% [43.80%, 45.36%]	n=9548<br>42.26% [41.79%, 42.73%]	1.21 [1.12, 1.30]	<0.001
**Intensity Modulated**	n=3318<br>36.99% [36.21%, 37.77%]	n=8419<br>37.26% [36.79%, 37.74%]	0.98 [0.91, 1.06]	0.58
**Proton**	n=45<br>0.5% [0.27%, 0.73%]	n=380<br>1.68% [1.54%, 1.82%]	0.26 [0.19, 0.38]	<0.001
**Stereotactic**	n=1257<br>14.01% [13.37%, 14.46%]	n=3369<br>14.91% [14.52%, 15.30%]	0.88 [0.80, 0.97]	0.013
**Brachytherapy**	n=339<br>3.78% [3.51%, 4.05%]	n=838<br>3.71% [3.54%, 3.87%]	1.04 [0.80, 1.35]	0.77
**Radiation Treatment Length (Number of Treatments)**				
**Overall**	21.29 [21.06, 21.51]	19.51 [19.37, 19.65]	1.08 [1.06, 1.10]	<0.001
**2D/3D Conventional**	17.43 [17.19, 17.67]	15.95 [15.81, 16.10]	1.08 [1.06, 1.10]	<0.001
**Intensity Modulated**	33.39 [33.06, 33.71]	30.54 [30.35, 30.74]	1.09 [1.07, 1.10]	<0.001
**Proton**	37.35 [35.24, 39.46]	31.80 [31.15, 32.45]	1.15 [1.07, 1.24]	<0.001
**Stereotactic**	4.31 [4.22, 4.41]	3.93 [3.88, 3.98]	1.10 [1.05, 1.14]	<0.001
**Brachytherapy**	7.51 [7.23, 7.80]	7.21 [7.05, 7.36]	1.05 [0.98, 1.13]	0.15
**Radiation Therapy Spending (Dollars)**				
**Overall**	$8724.63 [$8615.32, $8833.94]	$8374.51 [8308.15, $8440.88]	1.04 [1.02, 1.06]	<0.001
**2D/3D Conventional**	$4343.54 [$4275.64, $4411.43]	$3943.86 [$3902.98, $3984.75]	1.08 [1.06, 1.11]	<0.001
**Intensity Modulated**	$14,309.68 [$14,162.56, $14,456.80]	$13,222.20 [$13,131.81, $13,312.59]	1.09 [1.07, 1.11]	<0.001
**Proton**	$36,089 .29($34,007.65, $38,170.92]	$31,703.40 [$31,064.29, $32,342.51]	1.11 [1.01, 1.23]	0.037
**Stereotactic**	$8234.16 [$8071.09, $8397.24]	$7488.45 [$7400.08, $7576.83]	1.09 [1.06, 1.12]	<0.001
**Brachytherapy**	$3698.87 [$3545.54, $3852.20]	$3231.91 [$3151.08, $3312.74]	1.25 [1.17, 1.34]	<0.001

MA: Medicare Advantage, TM: Traditional Medicare, 2D: two-dimensional, 3D: three-dimensional, OR: Odds Ratio, RR: Rate Ratio, CI: Confidence Interval. Columns MA and TM contain predictive means from alternative linear regression models. County was adjusted for using fixed effects. Columns OR/RR (95% CI) contains the odds ratio/rate ratio with 95% CI using logistic regression when estimating the proportion of episodes using each treatment technology, a negative binomial model when estimating treatment length, and a log gamma regression model when estimating spending. For this analysis, covariates included insurance type (TM vs MA), cancer type, age, dual-eligibility status, patient comorbidities, county (fixed effects), and RT center type.

© 2025 Hogan J et al. *JAMA Network Open*.

eTable 5. Mean unadjusted number of treatments for all modalities and majority modality only

	MA	TM
	Mean N RT Treatments (Majority Modality Only)	
2D/3D Conventional	17.30	15.90
Intensity Modulated	33.51	29.95
Proton	36.88	31.46
Stereotactic	4.31	3.92
Brachytherapy	7.53	7.16
	Mean N RT Treatments (All Modalities)	
2D/3D Conventional	17.46	15.94
Intensity Modulated	33.98	30.29
Proton	37.18	31.83
Stereotactic	4.31	3.93
Brachytherapy	7.61	7.17

MA: Medicare Advantage, TM: Traditional Medicare, 2D: two-dimensional, 3D: three-dimensional, RT: radiotherapy

© 2025 Hogan J et al. *JAMA Network Open*.

eFigure 1. Radiotherapy technology type utilization across 90-day radiation therapy episodes overall, for MA vs TM, 2018

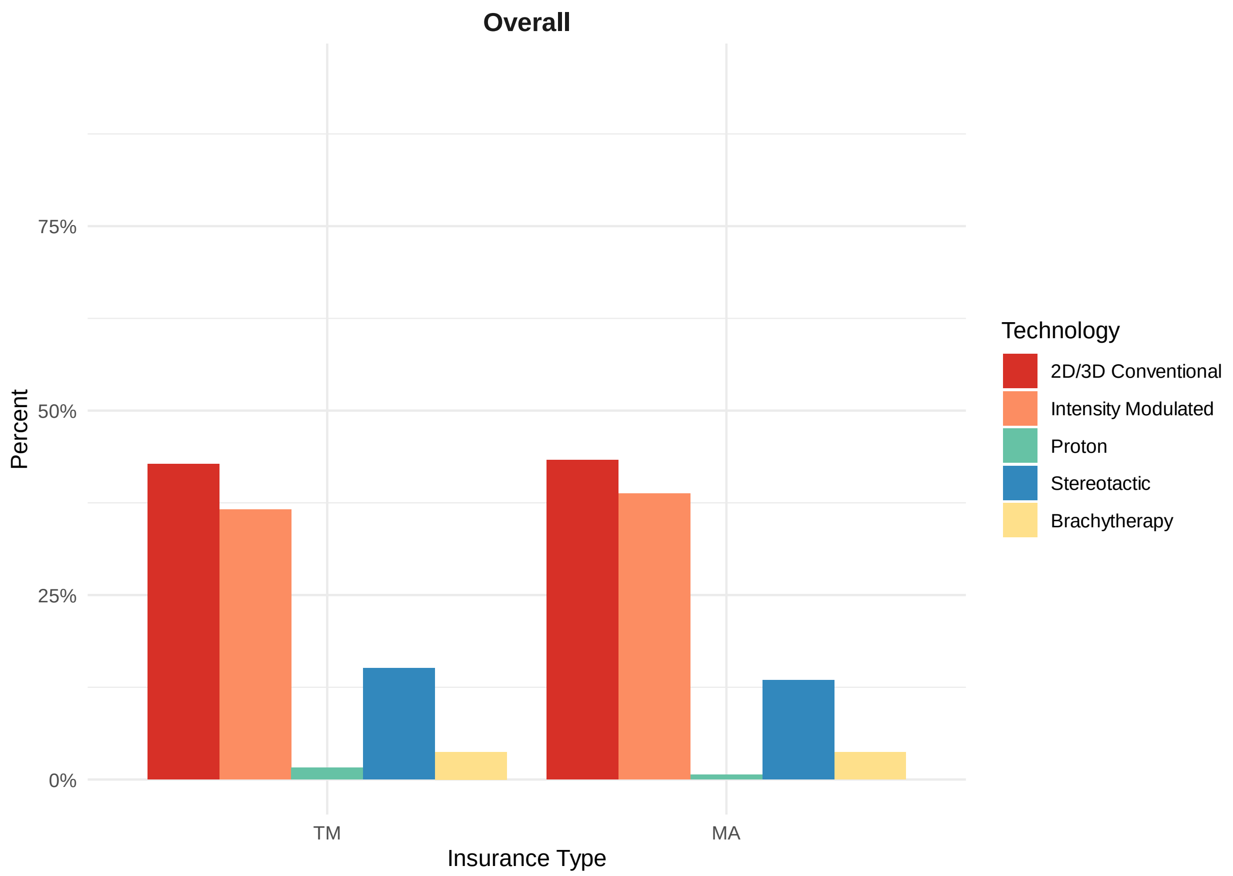

Bar chart showing proportion of episodes utilizing a certain type of radiation treatment technology. 2D, two-dimensional; 3D, three-dimensional; MA, Medicare Advantage; TM, Traditional Medicare.

© 2025 Hogan J et al. *JAMA Network Open*.

eFigure 2. Radiotherapy technology type utilization across 90-day radiation therapy episodes for anal cancer, MA vs TM, 2018

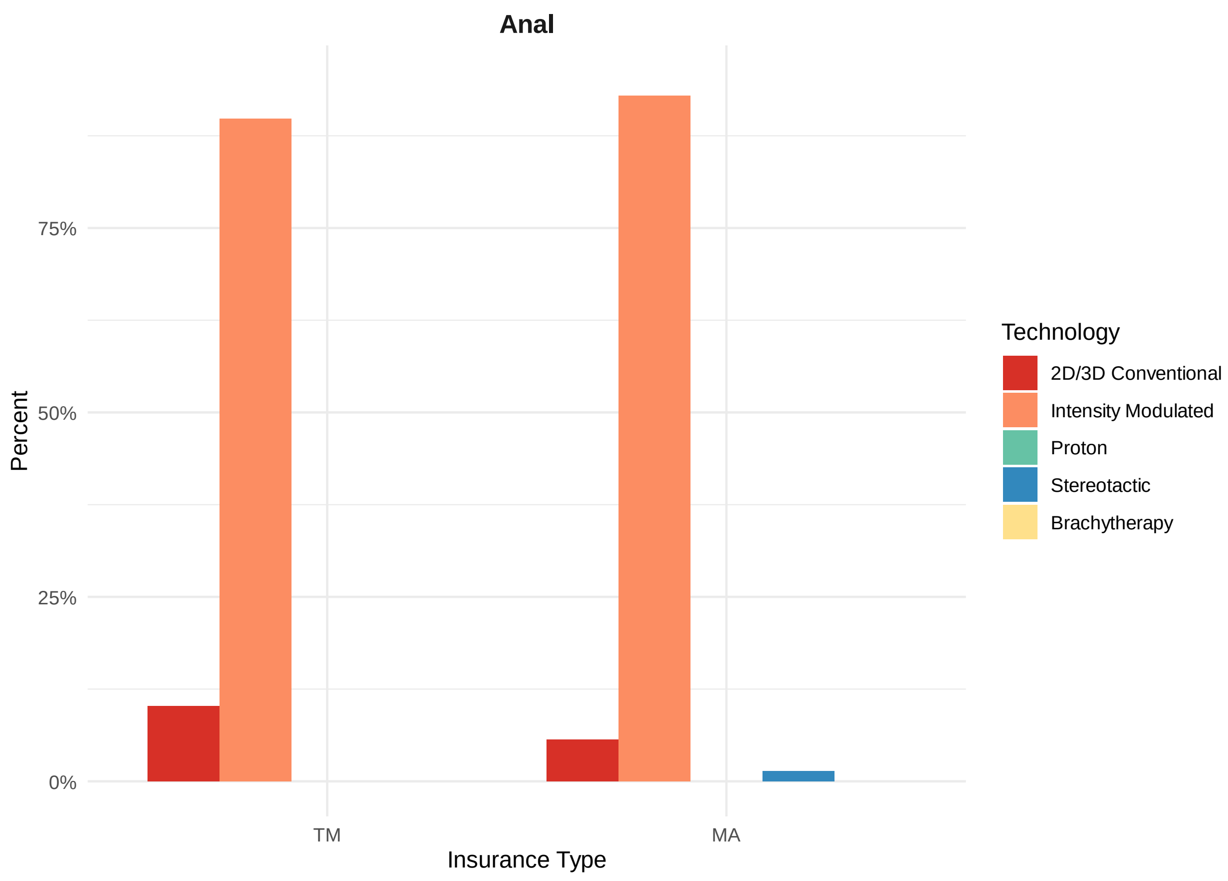

Bar chart showing proportion of episodes utilizing a certain type of radiation treatment technology. 2D, two-dimensional; 3D, three-dimensional; MA, Medicare Advantage; TM, Traditional Medicare.

© 2025 Hogan J et al. *JAMA Network Open*.

eFigure 3. Radiotherapy technology type utilization across 90-day radiation therapy episodes for bladder cancer, MA vs TM, 2018

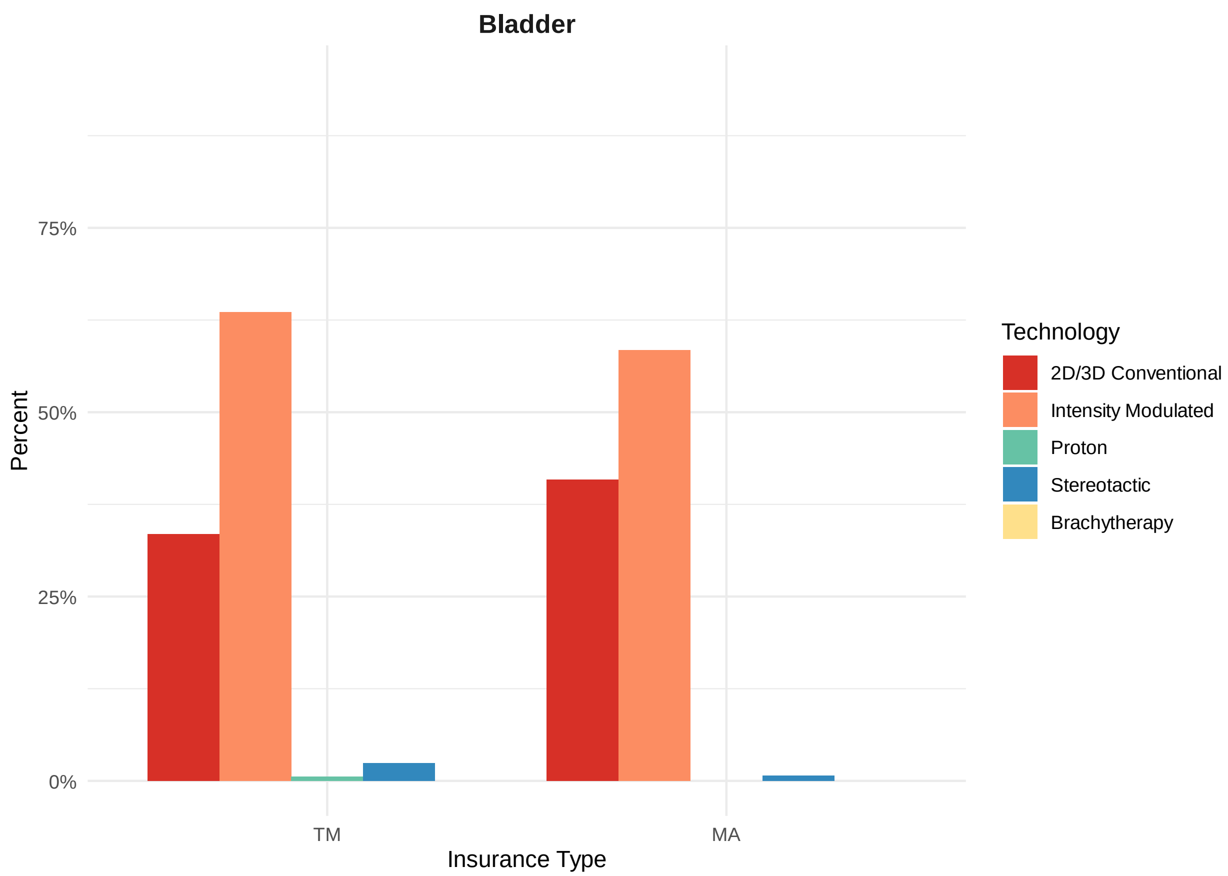

Bar chart showing proportion of episodes utilizing a certain type of radiation treatment technology. 2D, two-dimensional; 3D, three-dimensional; MA, Medicare Advantage; TM, Traditional Medicare.

© 2025 Hogan J et al. *JAMA Network Open*.

eFigure 4. Radiotherapy technology type utilization across 90-day radiation therapy episodes for bone metastases, MA vs TM, 2018

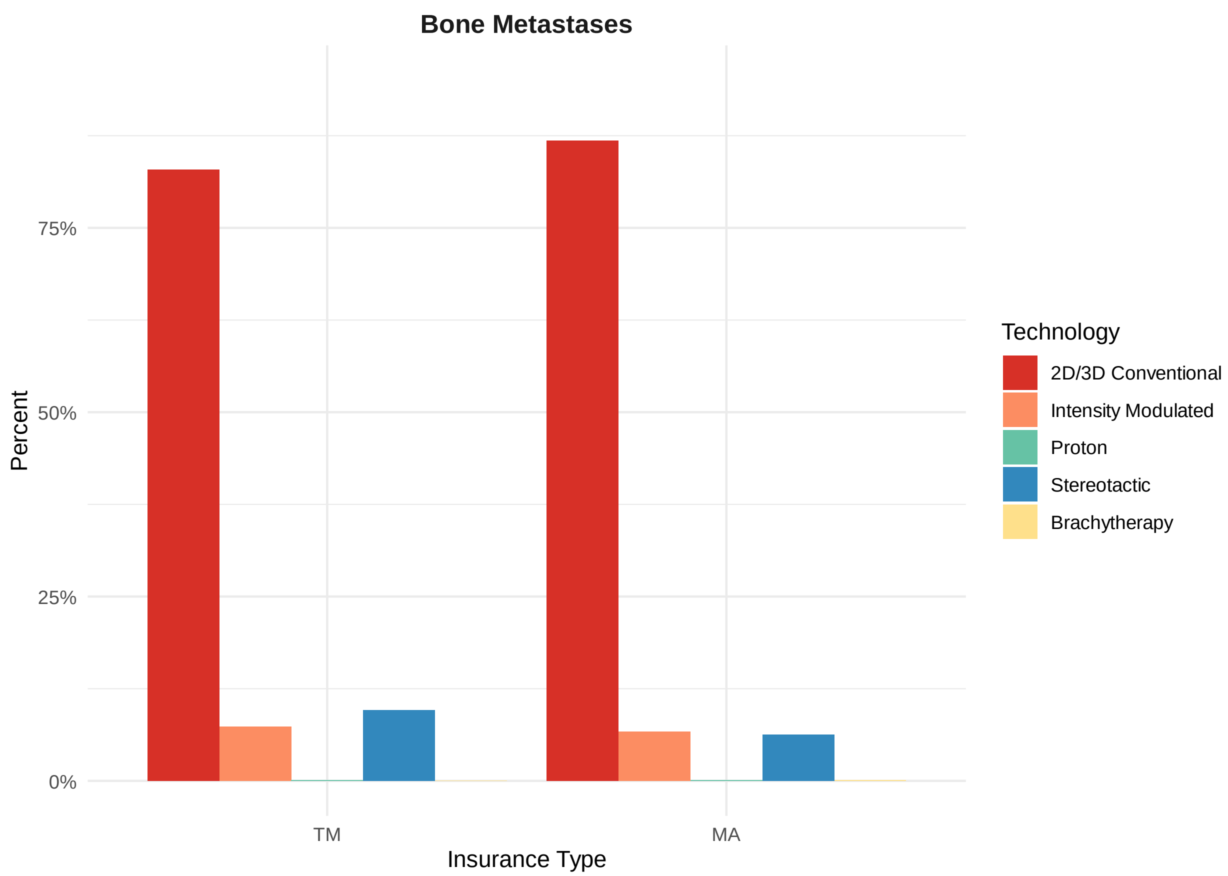

Bar chart showing proportion of episodes utilizing a certain type of radiation treatment technology. 2D, two-dimensional; 3D, three-dimensional; MA, Medicare Advantage; TM, Traditional Medicare.

© 2025 Hogan J et al. *JAMA Network Open*.

eFigure 5. Radiotherapy technology type utilization across 90-day radiation therapy episodes for brain metastases, MA vs TM, 2018

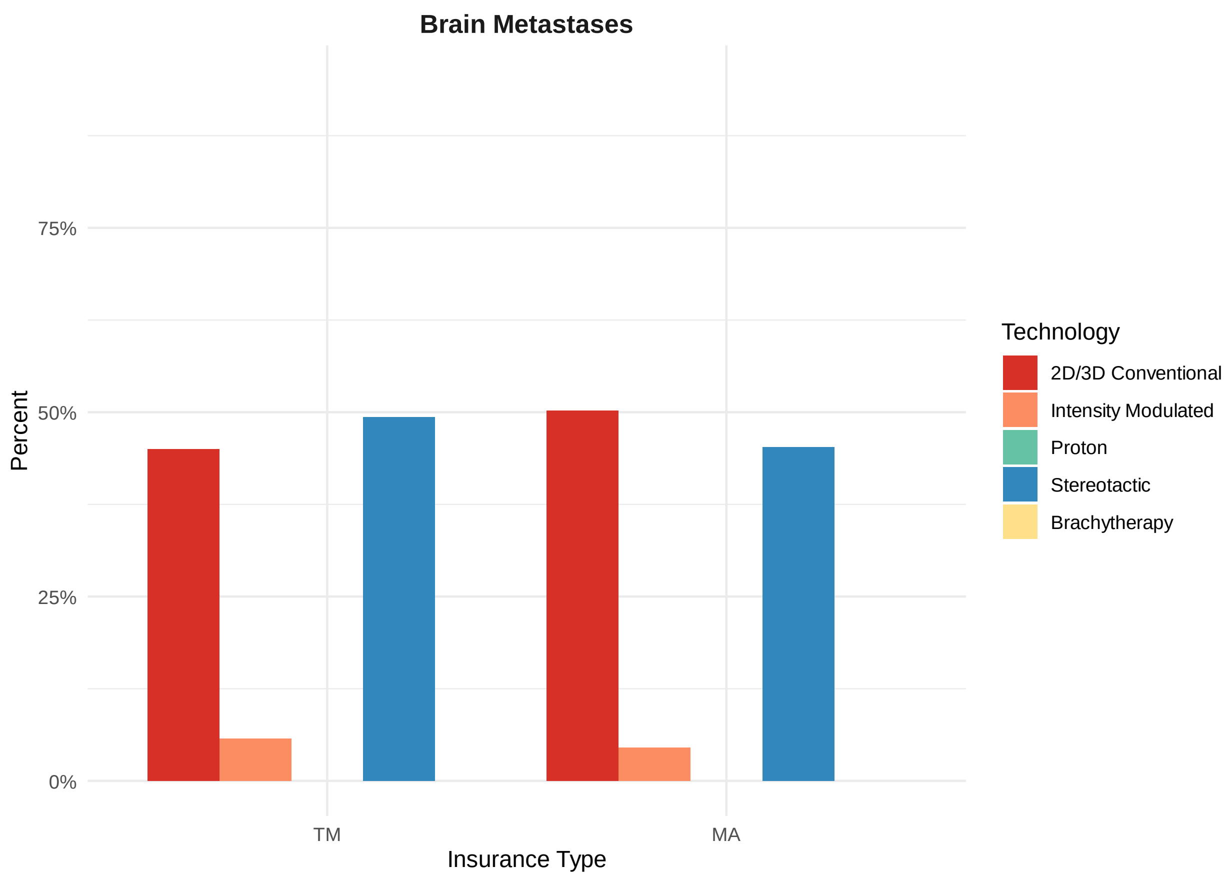

Bar chart showing proportion of episodes utilizing a certain type of radiation treatment technology. 2D, two-dimensional; 3D, three-dimensional; MA, Medicare Advantage; TM, Traditional Medicare.

© 2025 Hogan J et al. *JAMA Network Open*.

eFigure 6. Radiotherapy technology type utilization across 90-day radiation therapy episodes for breast cancer, MA vs TM, 2018

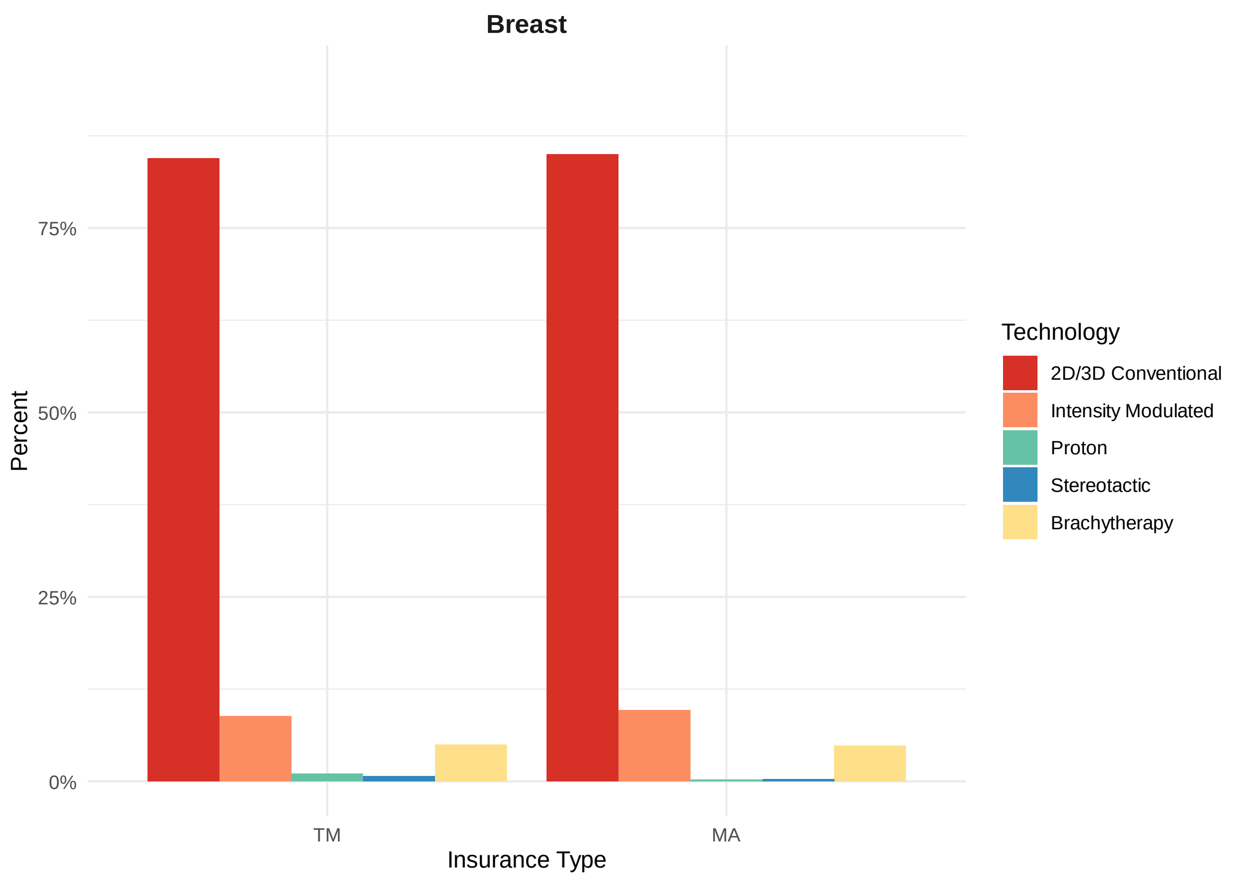

Bar chart showing proportion of episodes utilizing a certain type of radiation treatment technology. 2D, two-dimensional; 3D, three-dimensional; MA, Medicare Advantage; TM, Traditional Medicare.

© 2025 Hogan J et al. *JAMA Network Open*.

eFigure 7. Radiotherapy technology type utilization across 90-day radiation therapy episodes for cervical cancer, MA vs TM, 2018

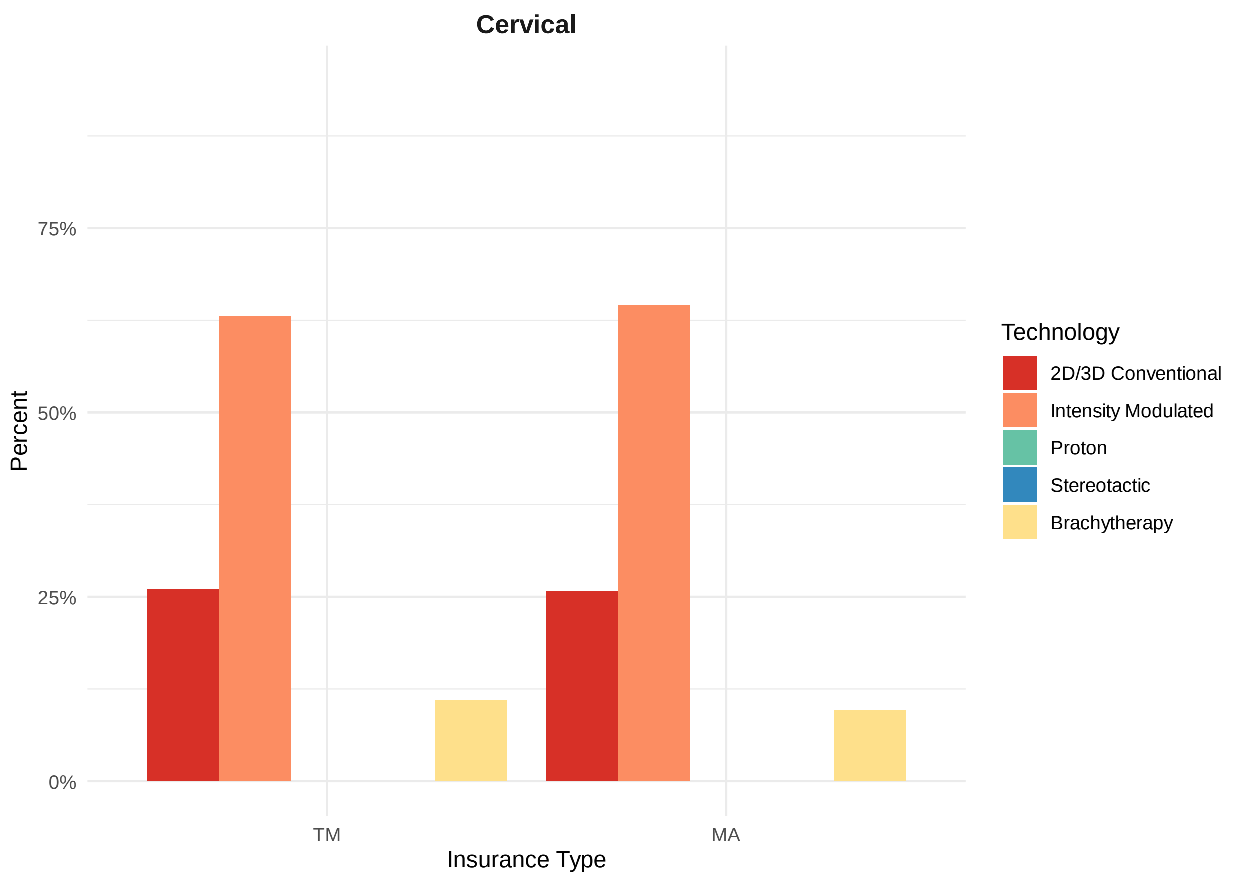

Bar chart showing proportion of episodes utilizing a certain type of radiation treatment technology. 2D, two-dimensional; 3D, three-dimensional; MA, Medicare Advantage; TM, Traditional Medicare.

© 2025 Hogan J et al. *JAMA Network Open*.

eFigure 8. Radiotherapy technology type utilization across 90-day radiation therapy episodes for CNS cancer, MA vs TM, 2018

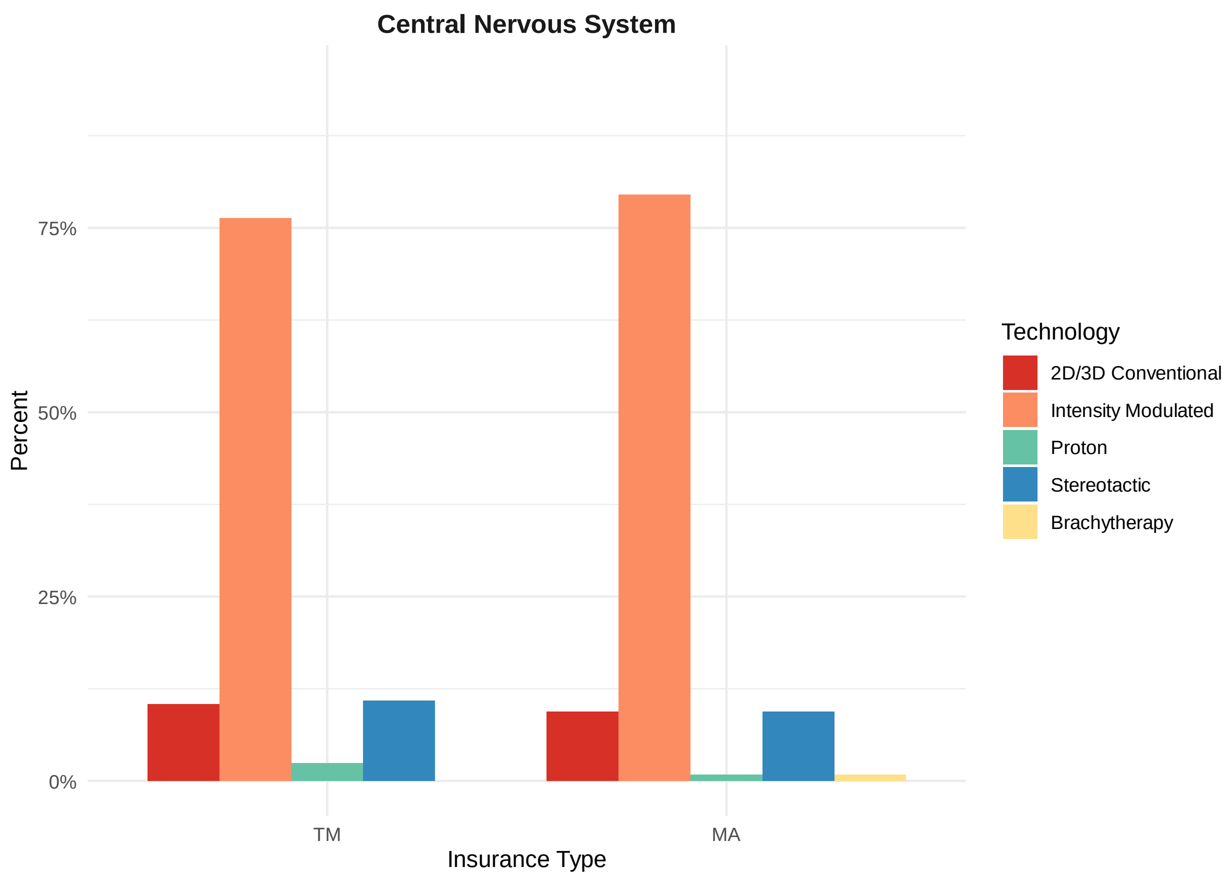

Bar chart showing proportion of episodes utilizing a certain type of radiation treatment technology. 2D, two-dimensional; 3D, three-dimensional; MA, Medicare Advantage; TM, Traditional Medicare; CNS, Central Nervous System.

© 2025 Hogan J et al. *JAMA Network Open*.

eFigure 9. Radiotherapy technology type utilization across 90-day radiation therapy episodes for colorectal cancer, MA vs TM, 2018

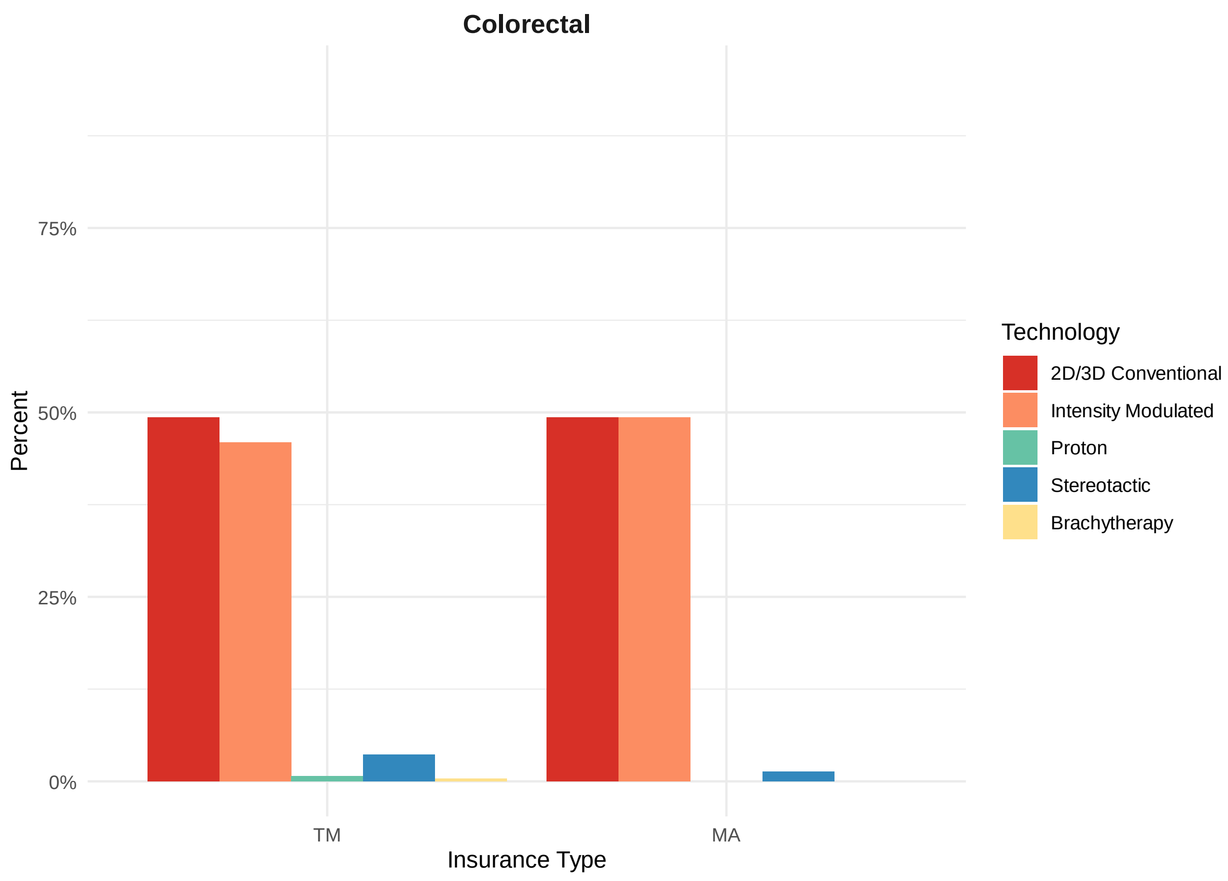

Bar chart showing proportion of episodes utilizing a certain type of radiation treatment technology. 2D, two-dimensional; 3D, three-dimensional; MA, Medicare Advantage; TM, Traditional Medicare.

© 2025 Hogan J et al. *JAMA Network Open*.

eFigure 10. Radiotherapy technology type utilization across 90-day radiation therapy episodes for head and neck cancer, MA vs TM, 2018

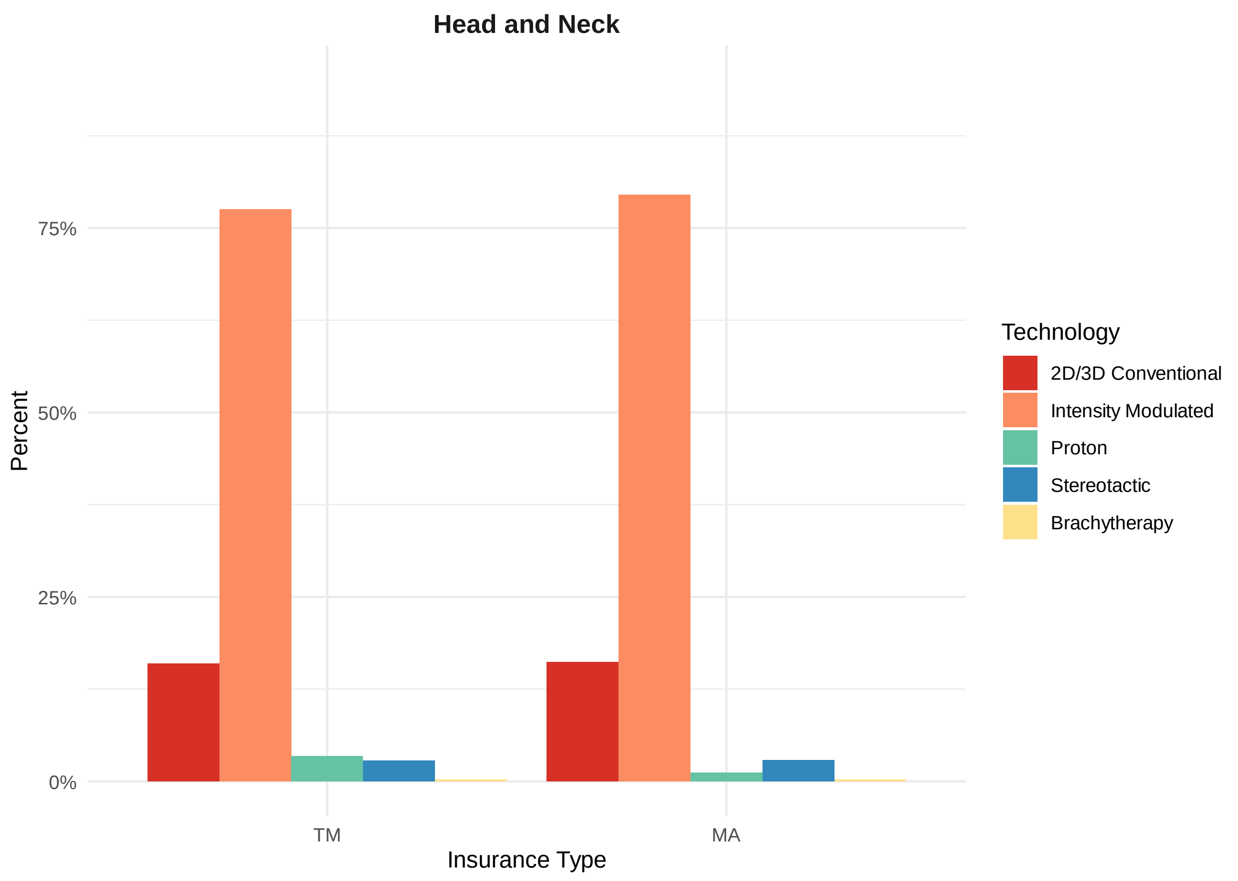

Bar chart showing proportion of episodes utilizing a certain type of radiation treatment technology. 2D, two-dimensional; 3D, three-dimensional; MA, Medicare Advantage; TM, Traditional Medicare; H&N, Head & Neck.

© 2025 Hogan J et al. *JAMA Network Open*.

eFigure 11. Radiotherapy technology type utilization across 90-day radiation therapy episodes for lung cancer, MA vs TM, 2018

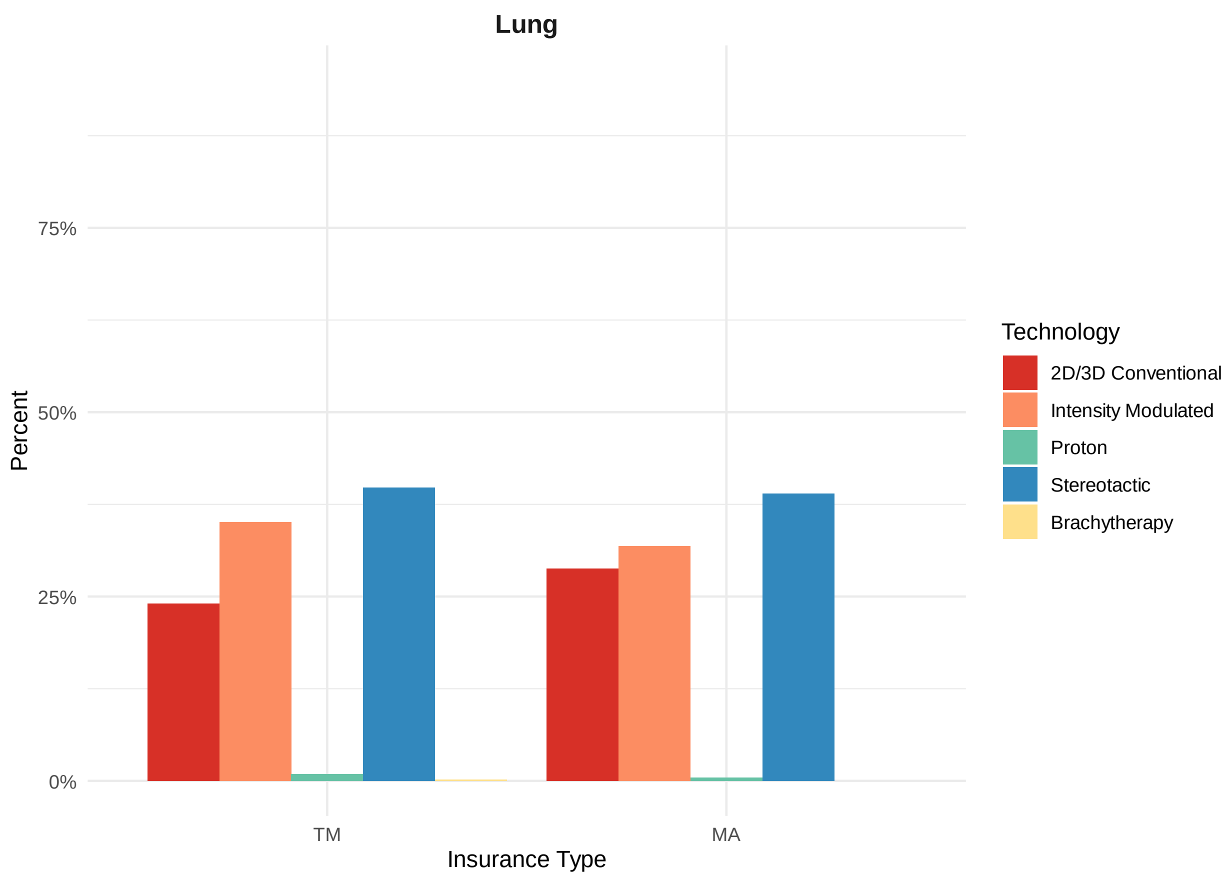

Bar chart showing proportion of episodes utilizing a certain type of radiation treatment technology. 2D, two-dimensional; 3D, three-dimensional; MA, Medicare Advantage; TM, Traditional Medicare.

© 2025 Hogan J et al. *JAMA Network Open*.

eFigure 12. Radiotherapy technology type utilization across 90-day radiation therapy episodes for lymphoma, MA vs TM, 2018

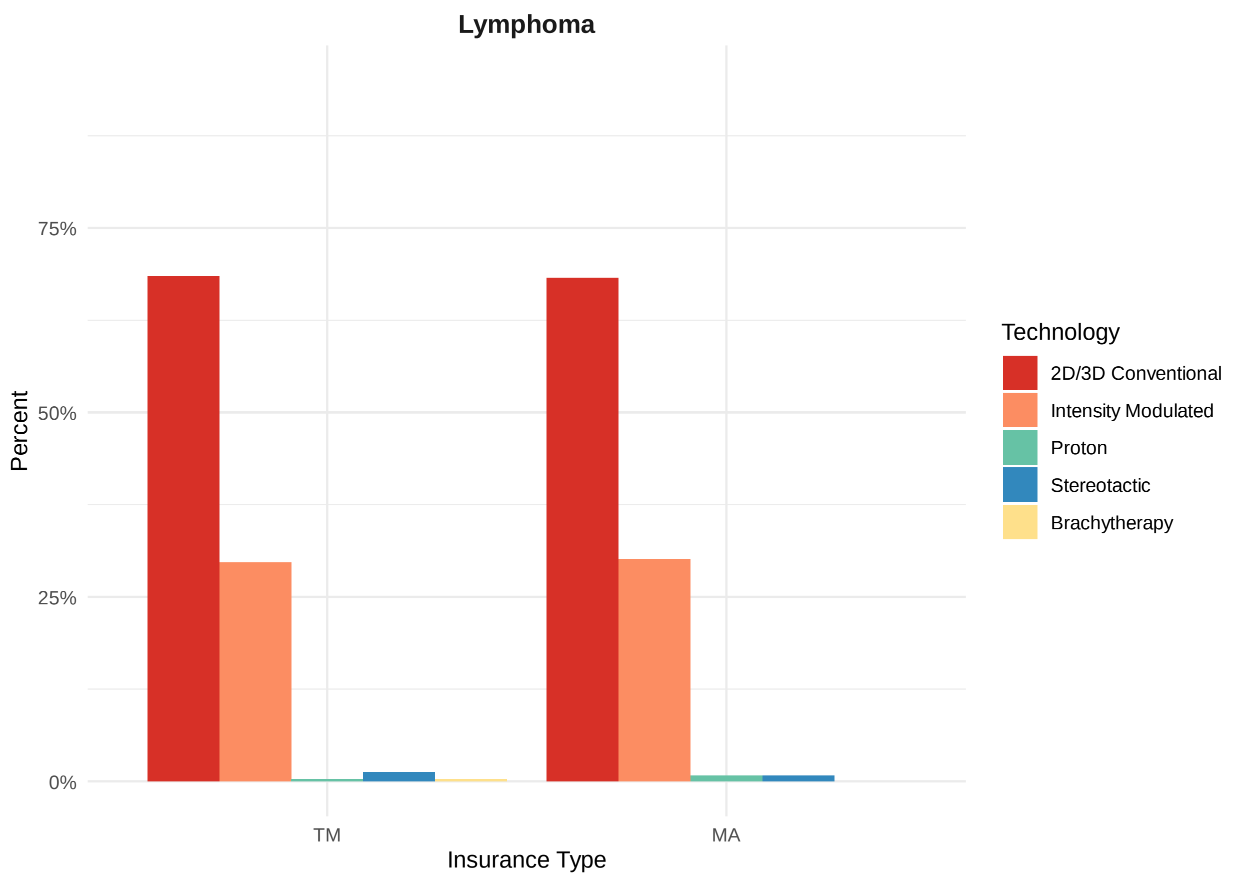

Bar chart showing proportion of episodes utilizing a certain type of radiation treatment technology. 2D, two-dimensional; 3D, three-dimensional; MA, Medicare Advantage; TM, Traditional Medicare.

© 2025 Hogan J et al. *JAMA Network Open*.

eFigure 13. Radiotherapy technology type utilization across 90-day radiation therapy episodes for pancreatic cancer, MA vs TM, 2018

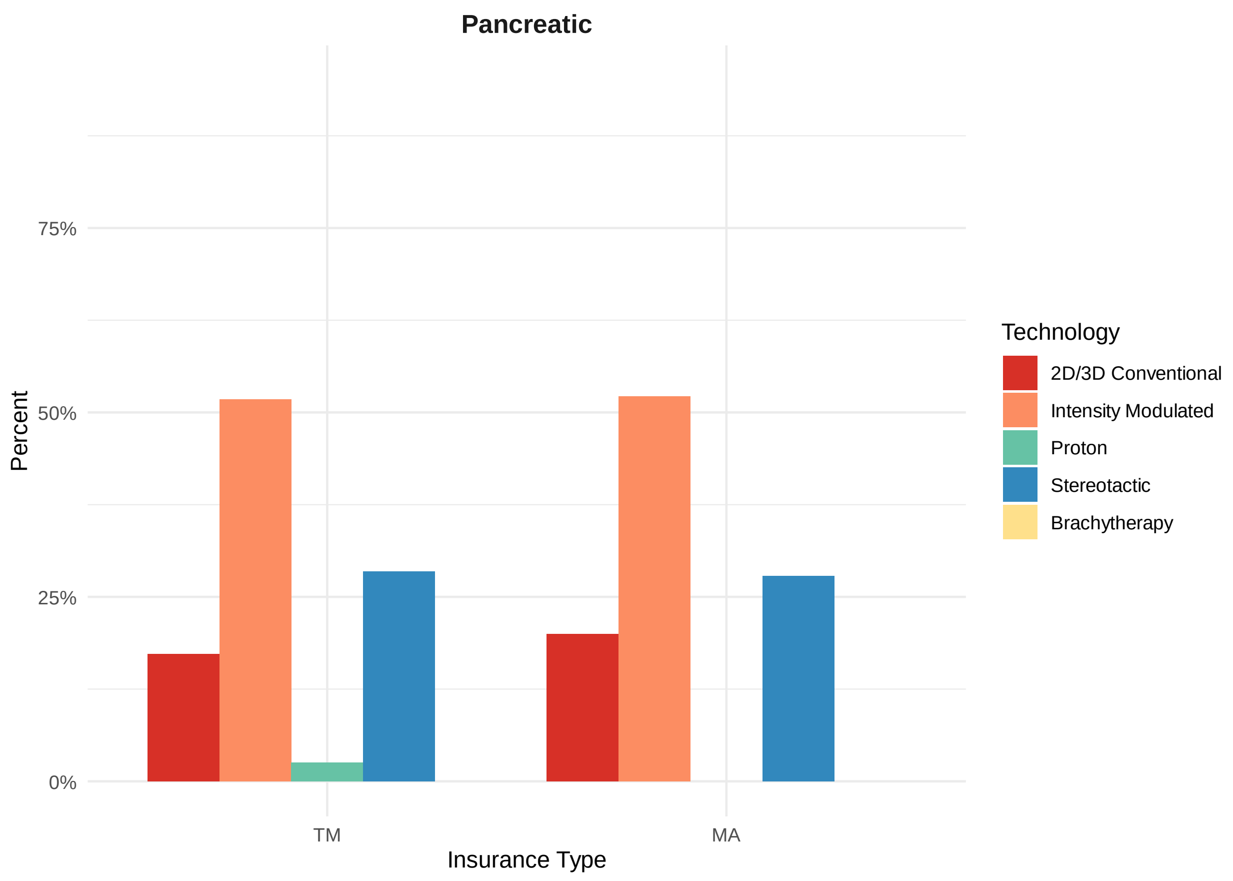

Bar chart showing proportion of episodes utilizing a certain type of radiation treatment technology. 2D, two-dimensional; 3D, three-dimensional; MA, Medicare Advantage; TM, Traditional Medicare.

© 2025 Hogan J et al. *JAMA Network Open*.

eFigure 14. Radiotherapy technology type utilization across 90-day radiation therapy episodes for prostate cancer, MA vs TM, 2018

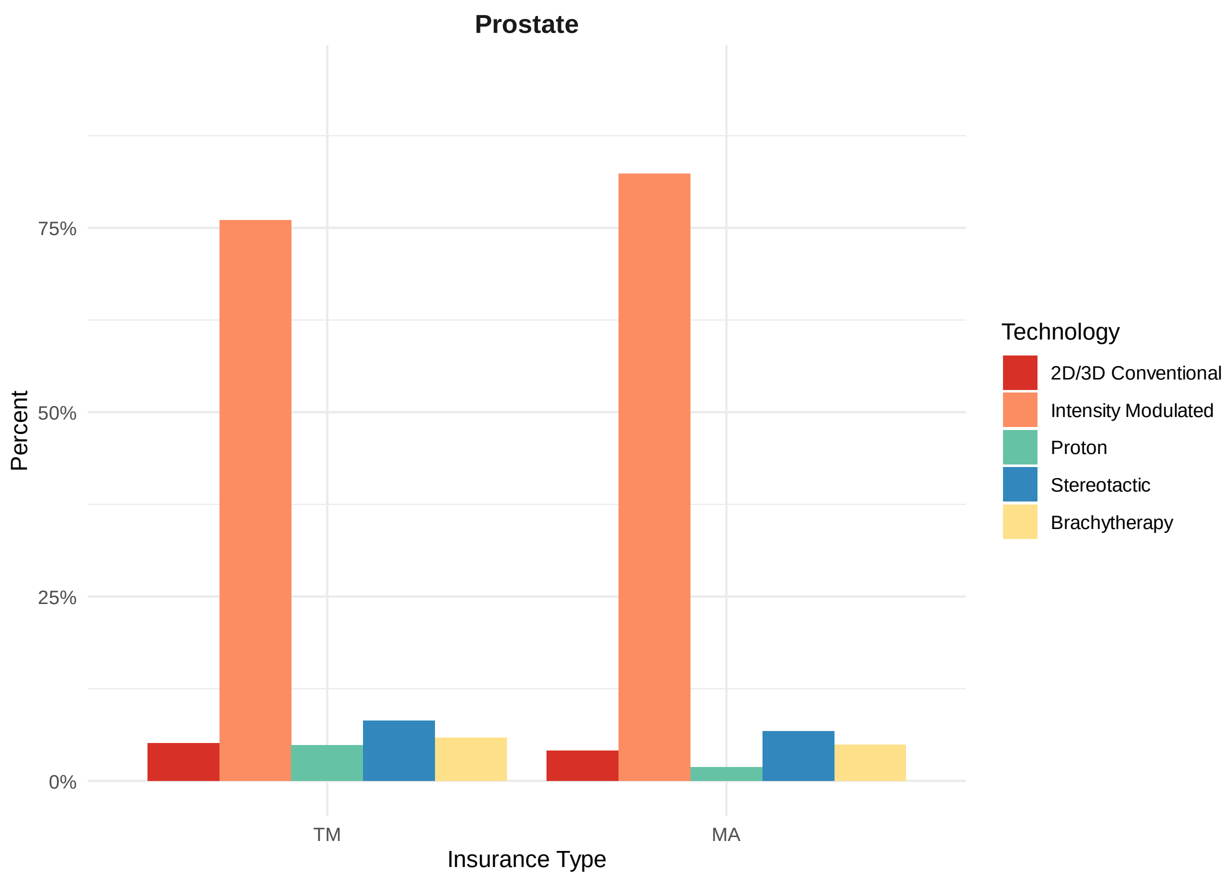

Bar chart showing proportion of episodes utilizing a certain type of radiation treatment technology. 2D, two-dimensional; 3D, three-dimensional; MA, Medicare Advantage; TM, Traditional Medicare.

© 2025 Hogan J et al. *JAMA Network Open*.

eFigure 15. Radiotherapy technology type utilization across 90-day radiation therapy episodes for upper gastrointestinal cancer, MA vs TM, 2018

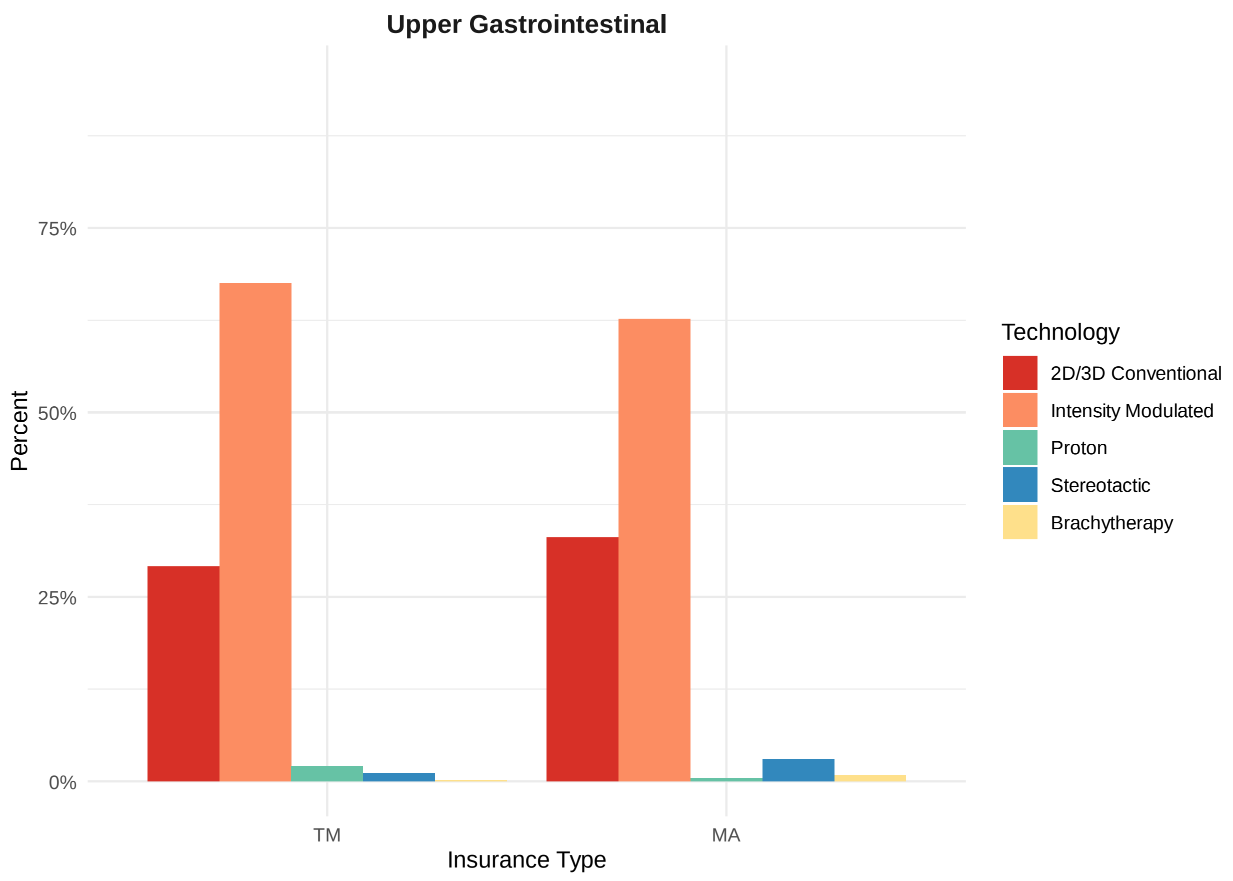

Bar chart showing proportion of episodes utilizing a certain type of radiation treatment technology. 2D, two-dimensional; 3D, three-dimensional; MA, Medicare Advantage; TM, Traditional Medicare.

© 2025 Hogan J et al. *JAMA Network Open*.

eFigure 16. Radiotherapy technology type utilization across 90-day radiation therapy episodes for uterine cancer, MA vs TM, 2018

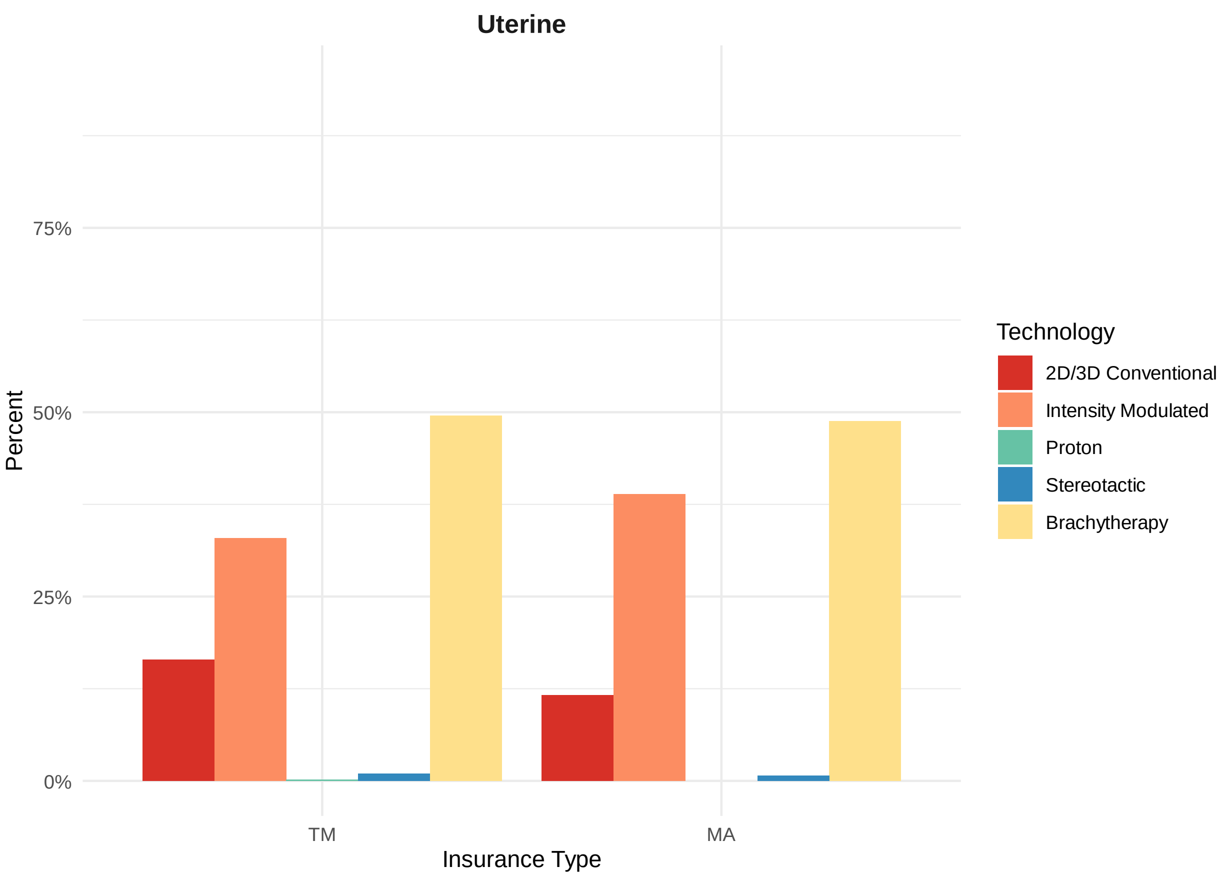

Bar chart showing proportion of episodes utilizing a certain type of radiation treatment technology. 2D, two-dimensional; 3D, three-dimensional; MA, Medicare Advantage; TM, Traditional Medicare.

© 2025 Hogan J et al. *JAMA Network Open*.
