# Supplementary material for: Radiotherapy Utilization in Traditional Medicare and Medicare Advantage
Source: JAMA Netw Open. 2025 Apr 2;8(4):e253018. doi: 10.1001/jamanetworkopen.2025.3018 (PMC11966333; doi:10.1001/jamanetworkopen.2025.3018)
Supplement: Supplement 2. — Data Sharing Statement [file jamanetwopen-e253018-s002.pdf]

# Data Sharing Statement

Hogan. Radiotherapy Utilization in Traditional Medicare and Medicare Advantage. *JAMA Netw Open*. Published April 02, 2025. doi:10.1001/jamanetworkopen.2025.3018

**Data**
**Data available:** No

**Additional Information**
**Explanation for why data not available:** Centers for Medicare and Medicaid do not allow for direct sharing of data without a Data Use Agreement.
